# Plausibility of the zebrafish embryos/larvae as an alternative animal model for autism: A comparison study of transcriptome changes

**DOI:** 10.1371/journal.pone.0203543

**Published:** 2018-09-04

**Authors:** Sangwoo Lee, Hang-Suk Chun, Jieon Lee, Han-Jin Park, Ki-Tae Kim, Cheol-Hee Kim, Seokjoo Yoon, Woo-Keun Kim

**Affiliations:** 1 System Toxicology Research Center, Korea Institute of Toxicology, Daejeon, Republic of Korea; 2 Predictive Model Research Center, Korea Institute of Toxicology, Daejeon, Republic of Korea; 3 Department of Environmental Engineering, Seoul National University of Science and Technology, Seoul, Republic of Korea; 4 Department of Biology, Chungnam National University, Daejeon, Republic of Korea; National Institutes of Health, UNITED STATES

## Abstract

Autism spectrum disorder (ASD) is a serious neurodevelopmental disorder characterized by impaired or abnormal social interaction and communication and by restricted and repetitive behaviour. ASD is highly prevalent in Asia, Europe, and the United States, and the frequency of ASD is growing each year. Recent epidemiological studies have indicated that ASD may be caused or triggered by exposure to chemicals in the environment, such as those in the air or water. Thus, toxicological studies are needed to examine chemicals that might be implicated. However, the experimental efficiency of existing experimental models is limited, and many models represent challenges in terms of animal welfare. Thus, alternative ASD animal models are necessary. To address this, we examined the efficacy of the zebrafish embryo/larva as an alternative model of ASD. Specifically, we exposed zebrafish to valproic acid (0, 12.5, 25, 50, or 100 μM), which is a chemical known to induce autism-like effects. We then analysed subsequent developmental, behavioural, and transcriptomic changes. We found that 100 μM and 50 μM valproic acid decreased the hatching rate and locomotor activity of zebrafish embryos/larvae. Transcriptomic analysis revealed significant alterations in a number of genes associated with autism, such as *adsl*, *mbd5*, *shank3*, and *tsc1b*. Additionally, we found changes in gene ontology that were also reported in previous studies. Our findings indicate that zebrafish embryos/larvae and humans with ASD might have common physiological pathways, indicating that this animal model may represent an alternative tool for examining the causes of and potential treatments for this illness.

## Introduction

Autism spectrum disorder (ASD) is an early onset neuropsychiatric disorder that is characterized by impaired social communication, restricted interests, and stereotyped and repetitive behaviours [1, 2]. Recent years have seen a dramatic increase in the prevalence of ASD in Asia, Europe, and the USA. The prevalence of ASD in Sweden (Stockholm cohort study) increased by almost 3.5-fold between 2001 and 2011 [3], and the prevalence in the United States increased from 1 in 150 children in 2002 to 1 in 68 children in 2012 [4]. The prevalence of ASD in South Korea has been found to be as high as 2.64% (95% CI = 1.91–3.37) [5]. Accordingly, the aetiology of ASD has been the subject of increasing focus [1, 2, 6–11]. Previous studies have described key genes and pathways associated with ASD that may be linked to synaptic scaffolding proteins, receptors, and cell adhesion molecules involved in chromatin remodelling, transcription, protein synthesis or degradation, and actin cytoskeleton dynamics [2]. Key genes involved in ASD are thought to include *eif4*, *GABA a receptor*, *mecp2*, *neurexin*, *neuroligins*, and *shank* [1, 2, 8, 12–14].

Although 54% of the rapid increase in ASD prevalence could be explained by diagnostic accretion, greater awareness, and increased parental age, the remaining portion of the increase is unexplained, indicating that an environmental agent may play a role [15]. Recently, epidemiological studies have produced evidence of a relationship between environmental pollutants and ASD [16–21]. A systematic review paper also showed that the aetiology of ASD may involve complex interactions between genetic factors and certain environmental toxicants [22]. For example, exposure to ambient air pollution and particulate matter with diameters ≤ 2.5 μm may be associated with ASD [16, 20]. Proximity to industrial facilities releasing arsenic, lead, or mercury has also been associated with ASD prevalence [18]. In addition, common agricultural pesticides (organophosphates and pyrethroids) and endocrine disrupting chemicals (polybrominated diphenyl ether-28 and *trans*-nonachlor) might increase the prevalence of ASD [17, 23]. Although children today are surrounded by thousands of synthetic chemicals, specific environmental chemicals that are linked to ASD prevalence have not been identified [24]. Few studies have examined the ASD-inducing effects of environmental chemicals, especially in terms of experimental toxicity. Furthermore, fewer than 20% of the high-volume chemicals for which epidemiological relationships with ASD have been reported have been tested for neurodevelopmental toxicity [24]. Therefore, experimental studies are required to examine the causal relationship between environmental chemical exposure and ASD.

To this end, experimental animal models are an indispensable tool [25]. Animal models for use in ASD research are mostly limited to rodents because of their robust behavioural phenotype and comprehensive genetic characterization [26, 27]. However, there is a growing need for models that are time and cost efficient, satisfy animal welfare criteria and are high throughput to screen the ASD-related toxicity of environmental chemicals and to identify effective pharmaceutical treatments for ASD from a massive chemical library [25]. The zebrafish (*Danio rerio*) is a new popular model organism in biomedical research [25]. The utility of both adult and larval zebrafish in neuroscience has grown markedly because it is a vertebrate species with high physiologic and genetic homology to humans and similar central nervous system (CNS) morphology [28]. Additionally, zebrafish have high fecundity (200–300 eggs per pair of adult fish) and produce a small-sized egg, which fits easily into a single well in a 96- or 384-well plate for high-throughput tests [29]. Thus, zebrafish are emerging as a new alternative model for ASD research [25, 28, 30]. However, transcriptome analysis and comparison studies focused on ASD have not yet been conducted with the zebrafish model [31–34].

Therefore, we designed the present study to evaluate the use of zebrafish as an alternative model for ASD research. We analysed developmental, behavioural, and transcriptomic changes and compared transcriptome data from zebrafish after exposure to valproic acid (VPA, an ASD inducer) with those from human ASD patients and a mouse model.

## Materials and methods

### Maintenance of zebrafish

Wild-type zebrafish (5D line) were generously provided by Robert L. Tanguay (Oregon State University, Corvallis, OR, USA) and maintained in a temperature-controlled room (25 ± 1°C) under a 14:10 h light:dark photoperiod. The zebrafish embryos used in the present study were obtained from mating pairs of adult zebrafish. The embryos were collected and examined under a stereomicroscope.

### Test chemical and exposure experiments

Valproic acid sodium salt (2-propylpentanoic acid sodium, CAS RN. 1069-66-5, purity > 98%) was purchased from Sigma-Aldrich (St. Louis, MO, USA). The test solutions were prepared in E3 medium, 0.292 g NaCl, 0.013 g KCl, 0.044 g CaCl, and 0.081 g MgSO_4_ in Millipore water (1 L). The exposure concentrations (0, 12.5, 25, 50, 100 μM of VPA) were determined according to previous studies [31, 34]. Normal zebrafish embryos were randomly distributed into test solutions of VPA for 120 h. All exposure experiments were conducted in accordance with protocols approved by the Institutional Animal Care and Use Committee (IACUC) of the Korea Institute of Toxicology (Protocol No. RS16005).

### Survival, hatching status, and malformation

During the exposure period, embryo/larva survival, hatching rate, time to hatch, and morphological malformation rate were checked every 24 h until 120 h. Percentages corresponding to mortality, hatching rate, and malformation rate were determined in quadruplicate (N = 4), with six embryos in each sample.

### Locomotor activity (movement tracking)

After 120 h exposure to VPA, we monitored the movement of each fish via an automated tracking device, Zebrabox™ (Viewpoint, Lyon, France). We tracked the larvae per treatment with octuplicates (N = 8) including three larvae in each. Tracking occurred during 4 repeated phases of a 3-min light/3-min dark period for a total of 24 min. The video data were then analysed using ZebraLab™ software to calculate the total distance travelled and active duration rate ((total movement duration–inactive duration) / total duration) for each individual larva.

### RNA-seq analysis

After 72 h and 120 h exposure, we pooled twenty zebrafish larvae per sample for RNA extraction. Triplicate (N = 3) pooled RNA samples from each treatment group were prepared and kept in RNA*later* RNA stabilization solution (Qiagen, Hilden, Germany) for further RNA extraction. Total RNA was isolated using the RNeasy mini kit (Qiagen, Hilden, Germany). We assessed rRNA band integrity using an Agilent RNA 6000 Nano kit (Agilent Technologies, CA, USA). We used samples with an RNA Integrity Number (RIN) greater than 7 for RNA library construction. Briefly, prior to cDNA library construction, we used 1 μg of total RNA and magnetic beads with oligo (dT) to enrich the poly (A) mRNA. Then, the purified mRNA was disrupted into short fragments, and the double-stranded cDNA was immediately synthesized. The cDNA was subjected to end-repair, poly (A) addition, and connected with sequencing adapters using the TruSeq RNA Sample Prep Kit (Illumina, Ca, USA). The suitable fragments, purified via a BluePippin 2% agarose gel cassette (Sage Science, MA, USA), were selected as templates for PCR amplification. The final libraries were quantified using a KAPA library quantification kit (KAPA Biosystems, South Africa), and the quality of the library was evaluated using an Agilent 2100 bioanalyser (Agilent Technologies, CA, USA). The fragments were found to contain between 350 and 450 base pairs. Subsequently, the library was sequenced using an Illumina HiSeq2500 sequencing platform (Illumina, CA, USA). Low-quality reads were filtered according to the following criteria: reads contained more than 10% skipped bases, reads contained more than 40% of bases whose quality scores are less than 20, and reads with average quality scores of each read less than 20. The filtered reads were mapped to the zebrafish reference genome (Ensembl version 86) using the aligner STAR v.2.3.0e [35].

### DEG analysis and GO analysis

We measured the gene expression level using Cufflinks v2.1.1 [36] with the gene annotation database from Ensembl version 86. The non-coding gene region was removed with the mask option. To improve the accuracy of measurement, we applied multi-read correction and frag bias correction. The abundance of gene transcripts was measured via FPKM (fragments per kilobase of transcript per million fragments mapped). The FPKM cut-off level was set at 0.1. For differential expression analysis, gene level count data were generated using the HTSeq-count v0.5.4p3 tool [37]. Using calculated read count data, differentially expressed genes (DEGs) were identified using the TCC R package [38]. Genes with a *p*-value < 0.05 and | log_2_FC(fold change) | > 1 were considered to be differentially expressed. Significantly altered DEGs were compared with the ASD-related gene lists from previous studies [2, 6, 9, 11, 39]. To predict the function of the selected genes, the DEGs were subjected to gene ontology (GO) according to three categories: biological process (BP), cellular component (CC), and molecular function (MF). The ontology and annotation files for GO enrichment analysis were downloaded from the gene ontology website (http://www.geneontology.org/). *p*-values < 0.001 were considered statistically significant [40]. Significantly altered GOs were compared with the ASD-related GO lists from previous studies [6, 41]. Concentration-dependent changes in GO enrichment were determined based on the gene lists showing transcriptional changes over 2 (| log_2_FC | > 1) between at least two serial concentrations with consistent positive or negative slopes.

### Quantitative PCR analysis

To validate the transcriptions of DEG analysis, quantitative PCR analysis was carried out. Target genes, i.e., *adsl* (*adenylosuccinate lyase*), *mbd5* (*methyl-CpG binding domain protein 5*), *shank3a* (*SH3 and multiple ankyrin repeat domains 3a*), and *tsc1b* (*tuberous sclerosis complex 1*), were chosen based on the commonality with previous studies (Table 1). After 120 h of VPA exposure, total RNA was extracted using a Maxwell 16 total RNA purification kit (Promega Co., Madison, WI, USA), and its concentration and quality were checked with an ND-1000 spectrophotometer (NanoDrop Technologies, Wilmington, ED, USA). During the RNA extraction, genomic DNA was selectively removed with the clearing agent that was included in the purification kit. cDNAs were synthesized by reverse transcription using an iScript cDNA synthesis kit (BioRad, Hercules, CA, USA). The cDNA concentrations were also measured using an ND-1000 spectrophotometer. The samples were then diluted with purified water to 300 ng/μL. Target genes were amplified using an ABI 7300 RT-PCR system (Applied Biosystems, Foster City, CA, USA). The PCR reaction mixture (total 20 μ/L) contained 10 μL of ABI SYBR Green Master mix (ABI, USA), 1.8 μL of each primer (10 pmol), 4.4 μL of purified PCR-grade water, and 2 μL of cDNA sample. The thermal cycle profile was as follows: pre-incubation at 95°C for 10 min, 40 cycles of amplification at 95°C for 10 s, 60°C for 20 s, and 72°C for 20 s. The comparative Ct methods were employed to calculate the relative level of gene transcription [42]. The sequence of primers for the target genes and reference gene (*β-actin*) for zebrafish are shown in S1 Table.

**Table 1:** Commonly observed ASD-related DEGs in this and previous studies.

Genes suggested by previous human studies						Description
Voineagu *et al*. (2011)	Pinto *et al*. (2014)	Sanders et al. (2015)	Bourgeron *et al*. (2015)^a)^		Chang *et al*. (2015)	
					*actn4*	alpha-actinin-4
***adsl***						**adenylosuccinate lyase**
		*capn12*				calcium-activate neutral proteinase 12
	*dcx (dcxr)*					dicarbonyl/L-xylulose reductase
					*ddb1*	damage-specific DNA binding protein 1
					*eif4a1* *(eif4a1a)*	eukaryotic translation initiation factor 4A1A
					*fanca*	fanconi anaemia group A protein
	*gamt*					guanidinoacetate N-methyltransferase
	*gatm*					glycine amidinotransferase (L-arginine:glycine amidinotransferase)
	*hsd17b10*					hydroxysteroid (17-beta) dehydrogenase 10
	*kcnj11*					potassium inwardly rectifying channel, subfamily J, member 11
	***mbd5***					**methyl-CpG binding domain protein 5**
					*myh11* *(myh11a)*	myosin, heavy chain 11a, smooth muscle
			*nrxn2*			neurexin-2
*pde9a*						high affinity cGMP-specific 3',5'-cyclic phosphodiesterase 9A
					*pfkm*	6-phosphofructokinase, muscle type
	*rpe65 (rpe65a)*					retinal pigment epithelium-specific protein 65a
*rpl10*						ribosomal protein L10
***shank3 (shank3a)***						**SH3 and multiple ankyrin repeat domains 3a**
		*slc6a1*				solute carrier family 6, member 1, like
*suclg2*						succinate-CoA ligase, GDP-forming, beta subunit
*tdo2*						tryptophan 2,3-dioxygenase a
		*trip12*				thyroid hormone receptor interactor 12
***tsc1(tsc1b)***			***tsc1 (tsc1b)***			**tuberous sclerosis 1b**

^a)^ Review article. DEGs were determined based on statistical significance (*p* < 0.05). Fold changes (log_2_FC) for each DEG are shown in S3–S7 Tables. DEGs from multiple studies are marked in bold.

### Statistical analysis

The normality and homogeneity of each variance were analysed using the Shapiro-Wilk test and Levene’s test. Then, we conducted one-way analyses of variance (ANOVA) with Dunnett’s test or T3 tests using SPSS 12 for Windows® (SPSS, Chicago, IL, USA) to determine significant differences between the control and VPA exposure groups. For non-parametric data, we used the Kruskal-Wallis test. Differences with *p*-values less than 0.05 were considered significant. For trend analysis, linear regression analysis was carried out, and the statistical significance of a linear trend in the slope was determined (*p* for trend).

## Results

### Effects of VPA on survival, hatching status, and development

**Fig 1:**
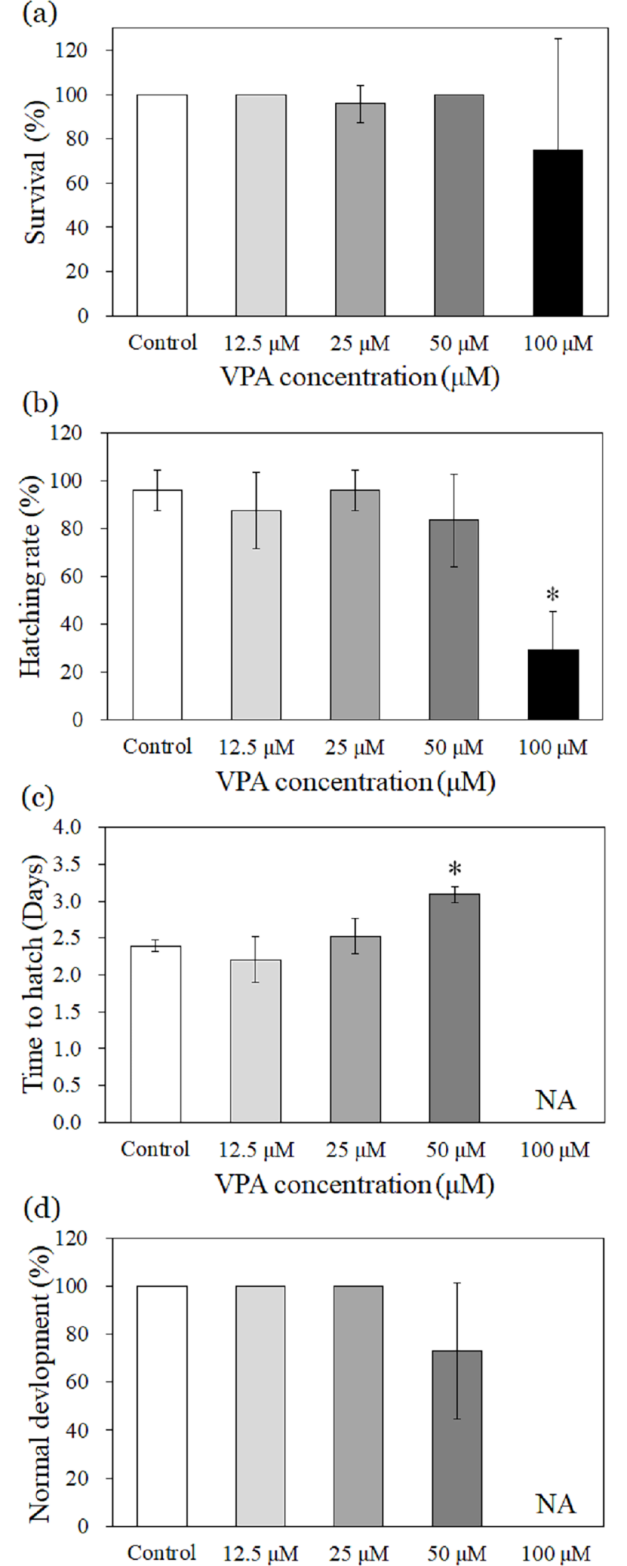
Effects of VPA on zebrafish survival, hatching status, and development. NA: not available due to significantly decreased hatching rate. The results are shown as the mean ± standard deviation (N = 4). Asterisk (*) denotes statistical significance (*p* < 0.05).

The survival of zebrafish larvae was unchanged for exposure up to 100 μM of VPA (Fig 1(A)). However, the hatching rate significantly decreased at 100 μM of VPA (Fig 1(B)) to only 29%. Time to hatch was significantly delayed at 50 μM of VPA (Fig 1(C)). We observed no significant developmental changes for exposure up to 50 μM of VPA (Fig 1(D)).

### Effects of VPA on larval behaviour

**Fig 2:**
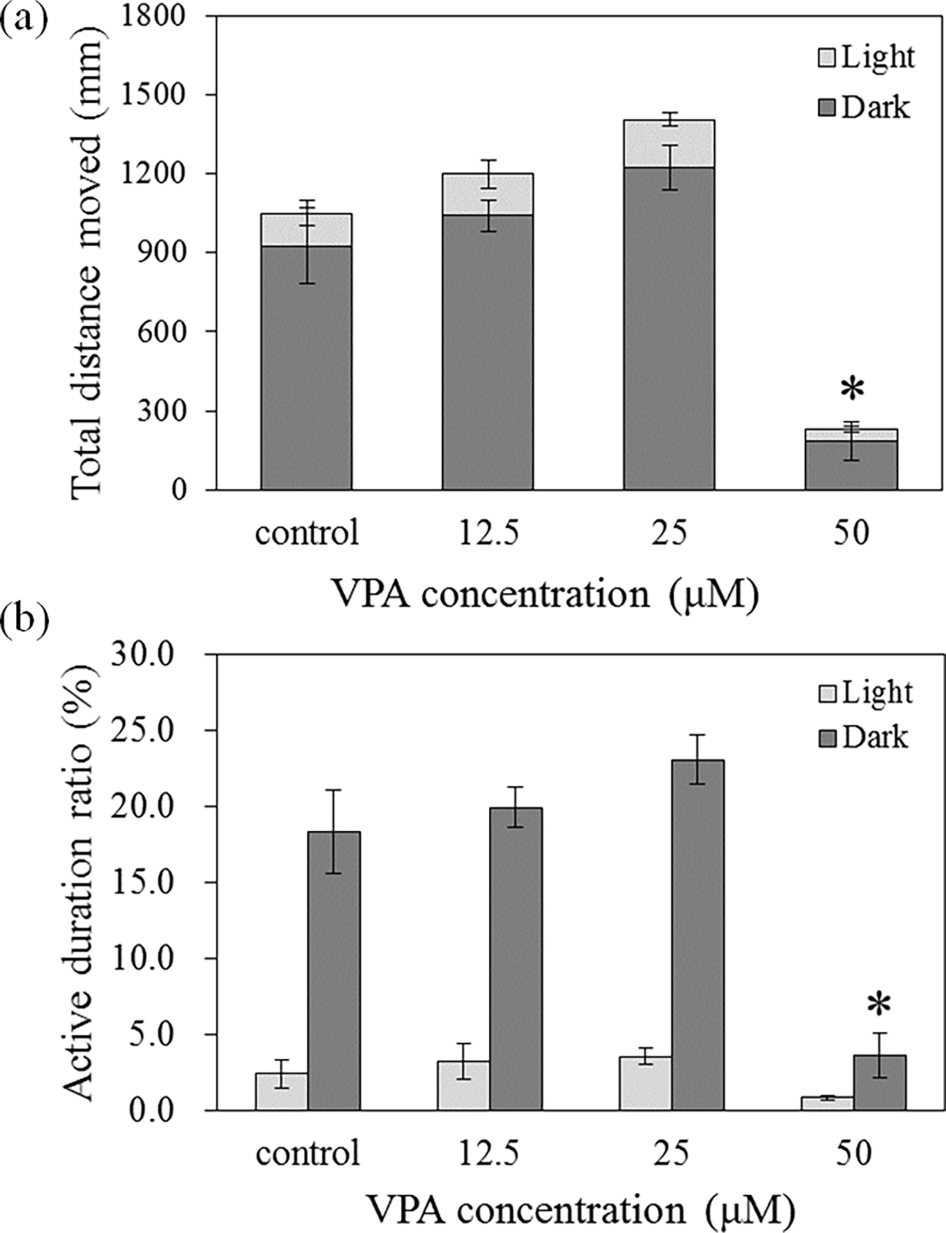
**Total distance moved (a) and active duration ratio (b) after 120 h VPA exposure. The error bars are marked separately for the dark and light phases.** The analysis the total distance moved was determined for the sum of the distance moved during the dark and light phases. The results are shown as the mean ± SEM (N = 8, except control (N = 7)). Asterisks (*) indicate statistical significance (*p* < 0.05).

VPA exposure at 50 μM caused a significant decrease in the total distance moved compared with control values (Fig 2(A)). At 50 μM VPA, the active duration ratio during the dark phase was significantly reduced to 20% of that in the control group (Fig 2(B)). However, at 25 μM VPA, the total distance moved and active duration ratio during the dark phase increased by 30% and 25%, respectively, although this was not statistically significant (Fig 2(A) and 2(B)).

### Illumina sequencing and mapping of RNA sequencing reads onto the zebrafish genome

High-throughput sequencing generated approximately 76.5–90.8 million (M) pairs of raw reads for each group (S2 Table). From each group, 75.5–89.8 M pairs of clean reads were extracted. Then, these high-quality reads were mapped to the reference zebrafish genome over 82.3%–88.3%. The uniquely mapped reads ranged from 78.8% to 85.3%.

### Analysis of differentially expressed zebrafish genes following VPA exposure

The number of DEGs ranged from 921 (12.5 μM at 72 h) to 1468 (25 μM at 120 h). The number of DEGs at 25 μM and 50 μM VPA were greater than those at 12.5 μM VPA at 72 h exposure (S1 Fig). At 120 h, the number of upregulated genes was greater than that of downregulated genes. However, there were more downregulated genes than upregulated genes at 72 h for all VPA conditions except 25 μM.

**Fig 3:**
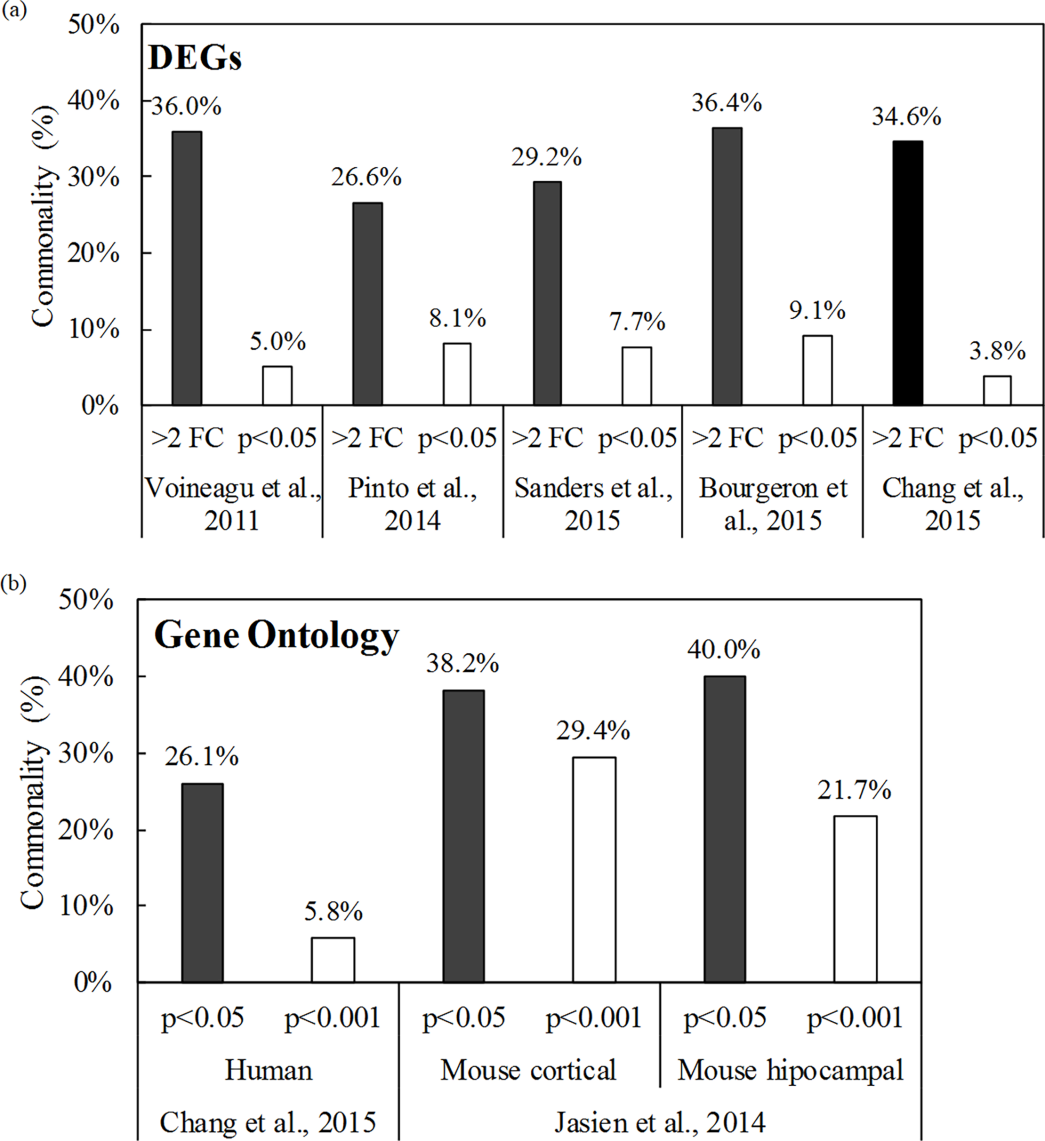
**Commonality of (a) DEGs and (b) GOs in the present study with respect to previous studies.** (a) Data from Voineagu *et al*. (2011), Pinto *et al*. (2014), Sanders *et al*. (2015), Bourgeron *et al*. (2015), and Chang *et al*. (2015). (b) Data from Chang *et al*. (2015) and Jasien *et al*. (2014).

Between 26.6% and 36.4% of the DEGs (> 2-fold change) in the present study matched ASD-related genes reported in previous studies [2, 6, 9, 11] (Fig 3(A)). Among them, 24 DEGs were significant (*p* < 0.05), as shown in Table 1. The transcription of genes indicated in previous studies to be ASD-related (e.g., *adsl*, *eif4a1* (*eukaryotic translation initiation factor 4a1*), *mbd5*, *nrxn2* (*neurexin2*), *shank3*, and *tsc1*, etc.) was significantly altered. The detailed data for DEGs that were significantly differentially expressed or expressed over 2-fold (| log2FC | > 1) in previous studies are shown in S3–S7 Tables.

**Fig 4:**
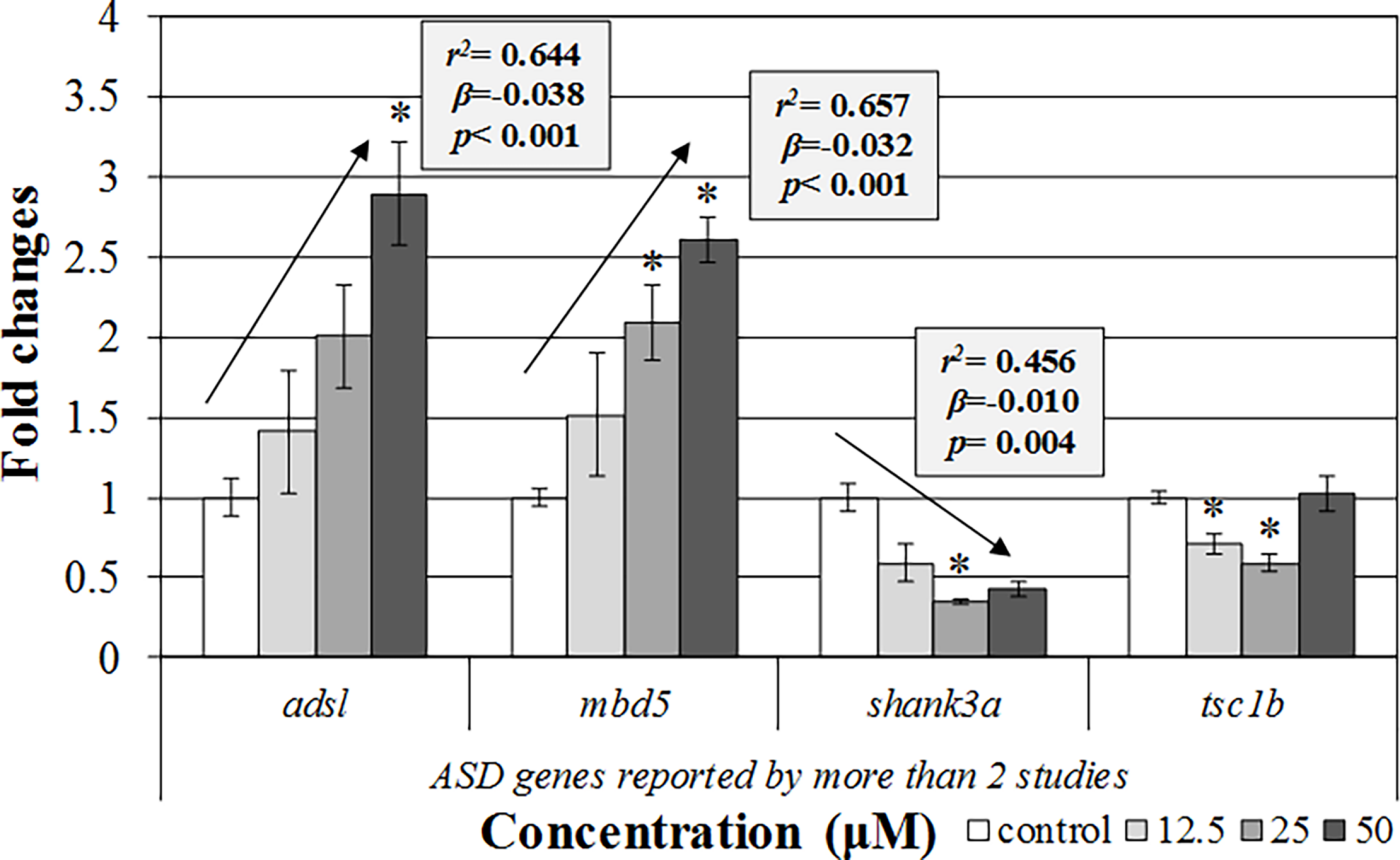
Gene transcription confirmation by using qRT-PCR analysis. The results are shown as the mean ± SEM (N = 4). Asterisks (*) and arrows indicate significant differences from the control and significant trend of the slope, respectively (*p* < 0.05).

The transcription of genes that had been commonly suggested as ASD-related genes by more than two previous studies and our study were significantly changed (Fig 4). Significant upregulation (*adsl* and *mbd5*) and downregulation (*shank3a* and *tsb1b*) were observed after 120 h VPA exposure in the qPCR analysis. Significant trends (*p* for trend) were also shown in the transcription of *adsl*, *mbd5a*, and *shank3a*.

### Analysis of GO enrichment in zebrafish after VPA exposure

In this study, we identified 44 GO enrichments that had been classified as ASD-related in previous studies [6, 41] (Table 2, S8 and S9 Tables). Half of the GOs were included in the CC category, and 13 and 9 of them were included in the MF and BP categories, respectively (Table 2). Significantly affected GO enrichments (*p* < 0.001) in this study were reported in 5.8% to 29.4% of previous human and mouse studies (Fig 3(B)). After VPA exposure, we found significant changes in GO enrichments related to transport (GO: 0006810), intracellular function (GO: 0005622), cytoplasm (GO: 0005737), mitochondria (GO: 0005739), cytosol (GO: 0005829), cytoskeleton (GO: 0005856), and cell projects (GO: 0042995), which are commonly found in the mouse cortex and hippocampus [41].

**Table 2:** Commonly observed GO enrichments in this and previous studies.

	Chang *et al*. (2015)^a)^	Jasien *et al*. (2014)^b)^		Term.
		Cortical	Hippocampal	
BP		GO:0006091		generation of precursor metabolites and energy
BP	GO:0006397			mRNA processing
BP		**GO:0006810**		**transport**
BP	GO:0006873			cellular ion homeostasis
BP	GO:0016071			mRNA metabolic process
BP		GO:0022904		respiratory electron transport chain
BP		GO:0044281		small molecule metabolic process
BP		GO:0045333		cellular respiration
BP	GO:0048729			tissue morphogenesis
CC		**GO:0005622**		**intracellular**
CC		**GO:0005737**		**cytoplasm**
CC		**GO:0005739**		**mitochondrion**
CC		GO:0005743		mitochondrial inner membrane
CC		GO:0005746		mitochondrial respiratory chain
CC		**GO:0005829**		**cytosol**
CC		**GO:0005856**		**cytoskeleton**
CC	GO:0005912			adherens junction
CC	GO:0005916			fascia adherens
CC	GO:0015629			actin cytoskeleton
CC	GO:0030016			myofibril
CC	GO:0030018			Z disc
CC	GO:0030027			lamellipodium
CC			GO:0030175	filopodium
CC	GO:0031252			cell leading edge
CC	GO:0031674			I band
CC		GO:0043229		intracellular organelle
CC		**GO:0042995**		**cell projection**
CC		GO:0043234		protein complex
CC	GO:0043292			contractile fibre
CC	GO:0070161			anchoring junction
CC		GO:0070469		respiratory chain
MF	GO:0003678			DNA helicase activity
MF	GO:0003774			motor activity
MF		GO:0003824		catalytic activity
MF		GO:0003954		NADH dehydrogenase activity
MF	GO:0004386			helicase activity
MF		GO:0008137		NADH dehydrogenase (ubiquinone) activity
MF		GO:0015078		hydrogen ion transmembrane transporter activity
MF		GO:0016462		pyrophosphatase activity
MF		GO:0016491		oxidoreductase activity
MF		GO:0016817		hydrolase activity, acting on acid anhydrides
MF		GO:0016818		hydrolase activity, acting on acid anhydrides, in phosphorus-containing anhydrides
MF		GO:0017111		nucleoside-triphosphatase activity
MF	GO:0046873			metal ion transmembrane transporter activity

^a)^ List of GOs related to human ASD from Chang *et al*. (2015).

^b)^ List of GOs in the aged BTBR mouse model from Jasien *et al*. (2014). GOs were determined based on statistical significance (*p* < 0.001). The *p*-value of each GO is shown in S8 and S9 Tables. GO enrichments from multiple studies are marked in bold.

We found that VPA concentration dependently altered the number of ASD-related GOs (Table 3). A total of 59 GOs were identified as ASD-related, including 25 GOs for the BP, 20 GOs for the CC, and 14 GOs for the MF. Synaptic transmission (GO: 0007268), transmission of nerve impulses (GO: 0019226), multicellular organismal signalling (GO: 0035637), and neurological system processes (GO: 0050877) were included in the BP category. Synapse-related GOs, i.e., synapses (GO: 0045202) and synapse parts (GO: 0044456), were in the CC category, and channel activity-related GOs, such as ion channel activity (GO: 0005216), cation channel activity (GO: 0005261), or ligand-gated ion channel activity (GO: 0015276), were found in the MF category.

**Table 3:** GOs that exhibited significant concentration-dependent alterations among the ASD-related GOs suggested by previous studies.

Category	Chang *et al*. (2015)^a)^	Jasien *et al*. (2014)^b)^		Term	*p*-value
		Cortical	Hippocampal		
BP	**GO:0003001**		**GO:0003001**	**generation of a signal involved in cell-cell signalling**	3.0e-5
BP		GO:0003008		system process	2.1e-11
BP	GO:0006325			chromatin organization	2.3e-4
BP		**GO:0006810**		**transport**	9.5e-7
BP			GO:0006836	neurotransmitter transport	6.2e-5
BP	GO:0006875			cellular metal ion homeostasis	4.6e-4
BP	GO:0007187			sarcolemma	1.0e-6
BP	GO:0007188			adenylate cyclase-modulating G-protein coupled receptor signalling pathway	2.6e-6
BP			GO:0007267	cell-cell signalling	1.0e-4
BP	**GO:0007268**		**GO:0007268**	**synaptic transmission**	7.4e-6
BP	GO:0007613			memory	1.6e-4
BP	GO:0016477			cell migration	3.5e-5
BP	**GO:0019226**			**transmission of nerve impulse**	4.1e-5
BP			GO:0023061	signal release	3.0e-5
BP	GO:0030030			cell projection organization	5.0e-4
BP	GO:0030705			cytoskeleton-dependent intracellular transport	7.7e-4
BP	GO:0030799			regulation of cyclic nucleotide metabolic process	3.1e-4
BP	GO:0030802			regulation of cyclic nucleotide biosynthetic process	2.7e-4
BP	GO:0030808			regulation of nucleotide biosynthetic process	2.7e-4
BP	GO:0030814			regulation of cAMP metabolic process	1.4e-4
BP	GO:0030817			regulation of cAMP biosynthetic process	1.2e-4
BP		**GO:0032940**		**secretion by cell**	1.7e-6
BP		**GO:0035637**		**multicellular organismal signalling**	2.2e-4
BP	GO:0040029			regulation of gene expression, epigenetic	2.8e-4
BP			GO:0044707	single-multicellular organism process	8.1e-4
BP		**GO:0046903**		**secretion**	1.3e-6
BP	GO:0048870			cell motility	1.6e-5
BP		**GO:0050877**		**neurological system process**	5.6e-13
BP		GO:0051179		localization	1.2e-5
BP		**GO:0051234**		**establishment of localization**	4.8e-7
BP	GO:0051674			localization of cell	1.6e-5
BP	GO:0055065			metal ion homeostasis	9.1e-4
BP	GO:0055082			cellular chemical homeostasis	2.9e-4
BP			GO:0065008	regulation of biological quality	4.6e-6
BP	GO:0071944			cell periphery	2.6e-16
CC	GO:0000785			chromatin	1.1e-7
CC			GO:0005623	cell	5.0e-4
CC		**GO:0005737**		**cytoplasm**	2.3e-5
CC		**GO:0005829**		**cytosol**	4.0e-5
CC		GO:0005856		cytoskeleton	4.9e-5
CC			GO:0016023	cytoplasmic membrane-bounded vesicle	1.4e-4
CC			GO:0031410	cytoplasmic vesicle	2.0e-4
CC			GO:0031982	vesicle	9.1e-5
CC			GO:0031988	membrane-bounded vesicle	9.3e-5
CC			GO:0032991	macromolecular complex	3.2e-5
CC	**GO:0042995**			**cell projection (at 72 h)**	7.2e-4
CC	**GO:0042995**			**cell projection (at 120 h)**	6.0e-4
CC			GO:0043226	organelle	1.8e-4
CC			GO:0043229	intracellular organelle	1.7e-4
CC		**GO:0044444**		**cytoplasmic part**	7.2e-7
CC			GO:0044446	intracellular organelle part	4.9e-7
CC	**GO:0044456**		**GO:0044456**	**synapse part**	2.7e-5
CC	**GO:0045202**		**GO:0045202**	**synapse**	7.0e-7
CC	GO:0070161			anchoring junction	1.6e-5
CC			GO:0070469	respiratory chain	2.7e-4
MF			GO:0003824	catalytic activity	4.1e-5
MF	GO:0004713			protein tyrosine kinase activity	6.3e-4
MF	GO:0005216			ion channel activity	5.0e-4
MF	GO:0005244			voltage-gated ion channel activity	3.2e-5
MF	GO:0005261			cation channel activity	6.9e-4
MF			GO:0015078	hydrogen ion transmembrane transporter activity	3.5e-4
MF	GO:0015276			ligand-gated ion channel activity	1.5e-6
MF			GO:0019905	syntaxin binding	1.6e-4
MF	GO:0022832			voltage-gated channel activity	4.0e-5
MF	GO:0022834			ligand-gated channel activity	1.5e-6
MF	GO:0022836			gated channel activity	9.9e-6
MF	GO:0022838			substrate-specific channel activity	5.8e-5
MF	GO:0022843			voltage-gated cation channel activity	5.7e-5
MF	GO:0046873			metal ion transmembrane transporter activity	1.0e-5

^a)^ List of GOs related to human ASD from Chang *et al*. (2015).

^b)^ List of GOs in the aged BTBR mouse model from Jasien *et al*. (2014). GO enrichments from multiple studies are marked in bold.

## Discussion

### Plausibility of zebrafish embryos/larvae as an ASD model based on transcriptome changes

We found a common transcriptional profile (DEGs and GOs) that was also found in previous ASD studies using human populations and mouse models. Thus, our transcriptome profile comparison data support the potential of zebrafish as an alternative animal model for ASD research. The zebrafish model has been proposed for ASD research based on behaviour phenotype [25, 33], and several ASD-related genes have been detected in the zebrafish [30]. However, this is the first comparison study to use transcriptome profiles. Our findings may be useful in the development of an alternative and rapid experimental model for screening ASD-related effects induced by environmental chemical exposure.

We observed 24 DEGs that had previously been suggested to be ASD-related genes in zebrafish embryos/larvae after VPA exposure (Table 1). A total of 136 ASD-related genes, presented in S3–S7 Tables, exhibited significant (*p* < 0.05) or a 2-fold increase in expression (| log2FC | > 1). These results indicate that zebrafish might, at least partly, have common genes and physiological pathways for ASD. The DEG (| log2FC | > 1) commonalities in our study were as high as 36.4% (Fig 3). We believe that this is meaningful because the commonalities among the previous studies [2, 6, 9, 11] ranged from 6.3% to 60.6% (data were not shown). Additionally, commonalities with ASD were generally higher than those with other diseases (e.g., oral cancer or skin irritation), which would not expect an association with VPA exposure (S2(A) Fig) [43, 44].

In our study, we found genes (*adsl*, *mbd5*, *shank3a*, and *tsc1b*) that had been reported to be ASD-related genes by multiple previous studies [2, 6, 9, 11]. The *adsl* gene is involved in the *de novo* purine biosynthesis pathway [45]. Previous studies reported that deficiency of the *adsl* gene might result in infantile seizures and autism [45, 46, 47]. Although the exact mechanisms are not yet clear, the possible mechanisms might include deficient synthesis of purine nucleotides, impairment of the purine nucleotide cycle, and a build-up of defective enzyme substrates [45]. The *mbd5* gene was thought to interact with myocyte enhancer factor 2c (MEF2C) in a complex bound to DNA [48]. Disruption of this complex due to *mbd5* mutation altered gene expression in neurogenesis and neurodevelopment [48]. In a cohort study, deletion of *mbd5* was also associated with autistic features [49]. The *shank3* gene, which encodes synaptic scaffolding protein, is important for spine morphogenesis and synaptic plasticity [1, 7, 32]. It might play a critical role in the normal development of neuronal connectivity because its disruption at a genetic level causes defects at striatal synapses and cortico-striatal circuits [50], as well as development and speech delays [1, 7], which might be associated with ASD. The *tsc1* gene is thought to be linked to ASD via perturbation of cytoskeletal dynamics and dendritic spine structure by inhibition of the mTOR signalling cascade, which plays a crucial role in synapse protein synthesis [1]. Deletion of *tsc1* in mice elicited a loss of cerebellar Purkinje cells and produced ASD-like behaviour, including abnormal social interactions and repetitive behaviours [51]. In our qPCR analysis, transcriptions of those genes were significantly altered (Fig 4). VPA, a chemical that induces ASD, led to a significant increase in the transcription of *adsl5* and *mbd5* but a significant decrease in the transcription of *shank3* and *tsc1b* in our zebrafish embryo/larval model. These results were comparable with our results from RNA-seq analysis. As an exception, the direction of *shank3a* transcription was different. Among the exposed groups, however, the transcription pattern of *shank3a* was comparable. Despite the exception, the other results were considerably reliable.

Various genetic pathways, such as cell adhesion molecules, ion channels, scaffolding protein, cytoskeleton, and signalling pathways, are implicated in ASD [1]. We found some of the genes included in those pathways (*nrxn2* (cell adhesion molecules), *cacna1e*, *scn1a* (ion channel), *shank3* (scaffolding protein), *actn4*, *gria3*, *grid1* (cytoskeleton), and *eif4a* (signalling pathway)) in our transcriptome analysis (S3–S7 Tables). Moreover, ASD-related genes such as *ak1* (*adenylate kinase 1*) and *ddb1* (*damage-specific DNA binding protein 1*) were included in the top 100 DEGs that we found in the 50 μM VPA-exposed group, based on the *p*-value (S10 and S11 Tables). Our DEG results imply that zebrafish might possess similar ASD genetic pathways to humans and other animal models.

We found many of the common GO enrichments that are components of the ASD pathway [6, 40]. Specifically, we reported 44 and 69 significantly altered GOs based on the transcription changes compared with the control group (Table 2) and concentration-dependent response (Table 3), respectively. The commonality was up to 40.0% and 29.4%, respectively, based on the *p*-values (*p* < 0.05 or *p* < 0.001). Those values were higher than the commonality with other diseases, i.e., oral cancer (8.7% and 1.0%, respectively) or dermatological disorder (19.2% and 1.9%, respectively) (S2(B) Fig) [52]. These comparison data of GO enrichments may support the idea that zebrafish, at least partly, share common physiology for ASD with other models.

We showed higher commonality with ASD than with other diseases, especially in the GO enrichment comparisons with stringent *p*-values (*p* < 0.001) (S2 Fig). However, commonality was relatively low in the DEG comparison. These results might be because GO enrichments represent a comprehensive approach. This implies that GO enrichments might adjust the bias from the transcriptions of certain genes. However, the results in S2 Fig are shown by a simple comparison among the respective lists of DEGs and GO enrichments, which could be due to methodological limitations. However, commonalities with other diseases, i.e., oral cancer or dermatological disorder, were observed in our study, although those were relatively weak. This might be expected because alterations of such basic pathways, such as the cytoskeleton or cell division, are also relevant to oral cancer or dermatological disorders [44, 52]. In addition, VPA is known to have the potential to cause other side effects, such as hepatotoxicity and metabolic derangements, as well as VPA being an ASD inducer [53].

Among the significantly affected GOs, GOs involved in the cellular component pathway were abundant (Table 2). The presence of GO enrichments related to mitochondria (*mitochondrion* (GO: 0005739), *mitochondrial inner membrane* (GO: 0005743), and *mitochondrial respiratory chain* (GO: 0005746)) and the cytoskeleton (*cytoskeleton* (GO: 0005856) and *actin cytoskeleton* (GO: 0015629)) were significant (*p* < 0.001) (Table 2). Recent studies have indicated that mitochondria dysfunction augments and disseminates brain abnormalities related to ASD [54, 55]. Interestingly, a significant proportion of ASD patients have concomitant diseases related to mitochondrial disease and abnormal energy generation [56]. The cytoskeleton is also important for the positioning of cell adhesion molecules and neurotransmitter receptors at the synapse [2] and is essential for maintaining synaptic function [14]. The *tsc1* gene, which we found to be significantly transcribed in our study, is related to the cytoskeleton pathway in ASD [1]. In addition, a previous study found neuronal signalling/cytoskeleton pathways containing *actin cytoskeleton* (GO: 0015629), *actin cytoskeleton organization* (GO: 0015629), and others to be major pathways of VPA-induced GOs in zebrafish when compared using the human functional network analysis [6] (S8 Table).

We found that significantly affected GOs were extended in a concentration-dependent manner (Table 3). We observed GO enrichments closer to the direct ASD pathway in VPA-exposed zebrafish. GOs in the nervous system, e.g., *synaptic transmission* (GO: 0007268), *transmission of nerve impulse* (GO: 0019226), and *neurological system process* (GO: 0050877), were involved. Additionally, GO enrichments of synapses (*synapse part* (GO: 0044456) and *synapse* (GO: 0045202)) for which dysfunction is crucial for ASD were significant [2]. Furthermore, many of the ion channel-related GOs, which play an important role in the aetiology of ASD by enhancing neuronal excitability [1], were also significant, e.g., *ion channel activity* (GO: 0005216), *cation channel activity* (GO: 0005261), and *ligand-gated channel activity* (GO: 0022834). These significant changes observed in GOs in our study imply that zebrafish, at least partly, share commonalties with humans or mice with respect to ASD aetiology. This might indicate that zebrafish could be applicable to ASD research, e.g., ASD-inducing environmental chemical screening or ASD drug discovery.

### Plausibility of zebrafish embryos/larvae as an ASD model based on behavioural changes

In addition to changes in gene transcription, the development of an experimental animal model for ASD requires strategies for behavioural observation. This is important because the symptoms of ASD are generally defined by behavioural characteristics, i.e., impaired social communication, repetitive behaviour, and cognitive deficits [25]. Previous studies have examined locomotor activity and social interaction behaviour in a transgenic monkey model [12]. Similar behavioural tests, such as social preference tests, the partition test, and the reciprocal social interaction test, have also been applied to rodent models [27, 57]. Stewart et al. [25] compared measurable behavioural endpoints in rodent and zebrafish models and suggested a number of behavioural testing methods that could be applied to adult zebrafish. These included the social preference test, social interaction test, shoaling assessment, mirror stimulation test, and stereotypic circling and swimming [25]. However, adult zebrafish are not as effective as embryos for screening the ASD-like effects of many kinds of chemicals. The test tools for social behaviour in the zebrafish larvae stage have not yet been completely developed. Therefore, instead of social behaviour, locomotor activity and anxiety have been used as markers of the ASD phenotype in ASD toxicity tests involving zebrafish embryos [32, 34, 58]. Thus, in the present study, we analysed locomotor activity in zebrafish larvae after VPA exposure as a behavioural endpoint.

We found that locomotor activity, i.e., the total distance moved and active duration, significantly decreased after exposure to 50 μM VPA (Fig 2). Previous studies reported similar levels of hypoactivity [31]. However, these effects, observed in both the current and previous studies, might be related to a VPA-induced delay in zebrafish hatching (Fig 1). We found that locomotor activity in zebrafish larvae slightly increased following exposure to 25 μM VPA. This was in accordance with a previous study and might indicate that VPA exposure causes anxiety in zebrafish larvae [34]. In a previous study, VPA exposure caused anxiety in larval zebrafish and resulted in social interaction deficits in adults [34]. Therefore, the observed effects on locomotor activity at the larval stage in our study could be linked to ASD-like behaviour. Although locomotor activity alone is not sufficient as a model of ASD in zebrafish larvae, this may be an important step, as such locomotor changes could reflect changes in basal activity elicited by ASD in terms of transcriptional alteration.

### Further research regarding the use of zebrafish as an ASD experimental model

In the present study, we used transcriptome analysis and behaviour observation to show that the zebrafish embryo/larva has potential as an alternative animal model for ASD research. However, further studies are required. First, multiple chemicals used in ASD models, known as inducers, should be examined in the zebrafish embryo/larvae model. It is necessary to confirm that the zebrafish embryo/larvae model responds appropriately when other types of ASD inducers are used. Second, genes that are common to multiple ASD models and those that are particularly sensitive to environmental exposure should be determined. This genetic information may be useful in developing transgenic zebrafish models for screening the ASD-like effects of environmental chemicals and new drug candidates for ASD. Third, tools for appropriate behavioural examination should be optimized for the zebrafish embryos/larvae model, including, for instance, those that involve locomotor analysis.

## Conclusion

In this study, we evaluated the plausibility of zebrafish embryos/larvae as an alternative animal model for ASD research. To this end, we exposed zebrafish embryos/larvae to VPA, examined the subsequent developmental and behavioural changes, and analysed transcriptional alterations by RNA-seq. We found behavioural and transcriptional changes that matched those from previous ASD studies using humans and rodent models. The results of this preliminary study indicate that the zebrafish embryo/larva has potential as an alternative experimental animal model for ASD toxicity tests and ASD drug discovery.

## Supporting information

S1 Fig Differentially expressed genes (DEGs) after exposure to VPA for 72 h and 120 h. (DOCX)

S2 Fig Comparison of commonalities of (a) DEGs and (b) GOs between ASD (shown in Fig 3) and other oral and skin-related diseases.
(DOCX)

S1 Table Primer sequences for qRT-PCR analysis used in this study. (DOCX)

S2 Table Statistics for read mapping. (DOCX)

S3 Table Differentially expressed genes after VPA exposure in zebrafish embryos/larvae among the ASD-related genes suggested by Voineagu *et al*. (2011). (DOCX)

S4 Table Differentially expressed genes after VPA exposure in zebrafish embryos/larvae among the ASD-related genes suggested by Pinto *et al*. (2014). (DOCX)

S5 Table Differentially expressed genes after VPA exposure in zebrafish embryos/larvae among the ASD-related genes suggested by Sanders *et al*. (2015). (DOCX)

S6 Table Differentially expressed genes after VPA exposure in zebrafish embryos/larvae among the ASD-related genes suggested by Bourgeron *et al*. (2014). (DOCX)

S7 Table Differentially expressed genes after VPA exposure in zebrafish embryos/larvae among the ASD-related genes suggested by Chang *et al*. (2014). (DOCX)

S8 Table Significantly affected GO after VPA exposure in zebrafish embryos/larvae among the ASD-related GO suggested by Chang *et al*. (2014). (DOCX)

S9 Table Significantly affected GO after VPA exposure in zebrafish embryos/larvae among the ASD-related GOs in the BTBR mouse model suggested by Jasien *et al*. (2014). (DOCX)

S10 Table The top 100 DEGs after 50 μM VPA exposure at 72 h based on *p*-value. (DOCX)

S11 Table The top 100 DEGs after 50 μM VPA exposure at 120 h based on *p*-value. (DOCX)

## References

[1] Banerjee S, Riordan M, Bhat MA. Genetic aspects of autism spectrum disorders: insights from animal models. Front Cell Neurosci. 2014;8:58 10.3389/fncel.2014.00058 ; PubMed Central PMCID: PMCPMC3932417. 24605088 PMC3932417

[2] Bourgeron T. From the genetic architecture to synaptic plasticity in autism spectrum disorder. Nat Rev Neurosci. 2015;16(9):551–63. 10.1038/nrn3992 . 26289574

[3] Idring S, Lundberg M, Sturm H, Dalman C, Gumpert C, Rai D, et al Changes in Prevalence of Autism Spectrum Disorders in 2001–2011: Findings from the Stockholm Youth Cohort. Journal of Autism and Developmental Disorders. 2015;45(6):1766–73. 10.1007/s10803-014-2336-y 25475364

[4] Christensen D, Baio J, Braun K. Prevalence and Characteristics of Autism Spectrum Disorder Among Children Aged 8 Years—Autism and Developmental Disabilities Monitoring Network, 11 Sites, United States, 2012. 2016. 10.15585/mmwr.ss6503a1 PMC7909709 27031587

[5] Kim YS, Leventhal BL, Koh YJ, Fombonne E, Laska E, Lim EC, et al Prevalence of Autism Spectrum Disorders in a Total Population Sample. American Journal of Psychiatry. 2011;168(9):904–12. 10.1176/appi.ajp.2011.10101532 . 21558103

[6] Chang J, Gilman SR, Chiang AH, Sanders SJ, Vitkup D. Genotype to phenotype relationships in autism spectrum disorders. Nat Neurosci. 2015;18(2):191–8. 10.1038/nn.3907 ; PubMed Central PMCID: PMCPMC4397214. 25531569 PMC4397214

[7] Durand CM, Betancur C, Boeckers TM, Bockmann J, Chaste P, Fauchereau F, et al Mutations in the gene encoding the synaptic scaffolding protein SHANK3 are associated with autism spectrum disorders. Nat Genet. 2007;39(1):25–7. 10.1038/ng1933 ; PubMed Central PMCID: PMCPMC2082049. 17173049 PMC2082049

[8] Gkogkas CG, Khoutorsky A, Ran I, Rampakakis E, Nevarko T, Weatherill DB, et al Autism-related deficits via dysregulated eIF4E-dependent translational control. Nature. 2013;493(7432):371–7. 10.1038/nature11628 ; PubMed Central PMCID: PMCPMC4133997. 23172145 PMC4133997

[9] Pinto D, Delaby E, Merico D, Barbosa M, Merikangas A, Klei L, et al Convergence of genes and cellular pathways dysregulated in autism spectrum disorders. Am J Hum Genet. 2014;94(5):677–94. 10.1016/j.ajhg.2014.03.018 ; PubMed Central PMCID: PMCPMC4067558. 24768552 PMC4067558

[10] State MW, Sestan N. Neuroscience. The emerging biology of autism spectrum disorders. Science. 2012;337(6100):1301–3. 10.1126/science.1224989 ; PubMed Central PMCID: PMCPMC3657753. 22984058 PMC3657753

[11] Voineagu I, Wang X, Johnston P, Lowe JK, Tian Y, Horvath S, et al Transcriptomic analysis of autistic brain reveals convergent molecular pathology. Nature. 2011;474(7351):380–4. 10.1038/nature10110 ; PubMed Central PMCID: PMCPMC3607626. 21614001 PMC3607626

[12] Liu Z, Li X, Zhang JT, Cai YJ, Cheng TL, Cheng C, et al Autism-like behaviours and germline transmission in transgenic monkeys overexpressing MeCP2. Nature. 2016;530(7588):98–102. 10.1038/nature16533 . 26808898

[13] Mathur P, Guo S. Use of zebrafish as a model to understand mechanisms of addiction and complex neurobehavioral phenotypes. Neurobiol Dis. 2010;40(1):66–72. 10.1016/j.nbd.2010.05.016 ; PubMed Central PMCID: PMCPMC3021971. 20493262 PMC3021971

[14] Yoo H. Genetics of Autism Spectrum Disorder: Current Status and Possible Clinical Applications. Exp Neurobiol. 2015;24(4):257–72. 10.5607/en.2015.24.4.257 ; PubMed Central PMCID: PMCPMC4688327. 26713075 PMC4688327

[15] Weintraub K. The prevalence puzzle: Autism counts. Nature. 2011;479(7371):22–4. 10.1038/479022a . 22051656

[16] Becerra TA, Wilhelm M, Olsen J, Cockburn M, Ritz B. Ambient air pollution and autism in Los Angeles county, California. Environ Health Perspect. 2013;121(3):380–6. 10.1289/ehp.1205827 ; PubMed Central PMCID: PMCPMC3621187. 23249813 PMC3621187

[17] Braun JM, Kalkbrenner AE, Just AC, Yolton K, Calafat AM, Sjodin A, et al Gestational exposure to endocrine-disrupting chemicals and reciprocal social, repetitive, and stereotypic behaviors in 4- and 5-year-old children: the HOME study. Environ Health Perspect. 2014;122(5):513–20. 10.1289/ehp.1307261 ; PubMed Central PMCID: PMCPMC4014765. 24622245 PMC4014765

[18] Dickerson AS, Rahbar MH, Han I, Bakian AV, Bilder DA, Harrington RA, et al Autism spectrum disorder prevalence and proximity to industrial facilities releasing arsenic, lead or mercury. Sci Total Environ. 2015;536:245–51. 10.1016/j.scitotenv.2015.07.024 ; PubMed Central PMCID: PMCPMC4721249. 26218563 PMC4721249

[19] Kalkbrenner AE, Schmidt RJ, Penlesky AC. Environmental chemical exposures and autism spectrum disorders: a review of the epidemiological evidence. Curr Probl Pediatr Adolesc Health Care. 2014;44(10):277–318. 10.1016/j.cppeds.2014.06.001 ; PubMed Central PMCID: PMCPMC4855851. 25199954 PMC4855851

[20] Raz R, Roberts AL, Lyall K, Hart JE, Just AC, Laden F, et al Autism spectrum disorder and particulate matter air pollution before, during, and after pregnancy: a nested case-control analysis within the Nurses' Health Study II Cohort. Environ Health Perspect. 2015;123(3):264–70. 10.1289/ehp.1408133 ; PubMed Central PMCID: PMCPMC4348742. 25522338 PMC4348742

[21] Shelton JF, Hertz-Picciotto I, Pessah IN. Tipping the balance of autism risk: potential mechanisms linking pesticides and autism. Environ Health Perspect. 2012;120(7):944–51. 10.1289/ehp.1104553 ; PubMed Central PMCID: PMCPMC3404662. 22534084 PMC3404662

[22] Rossignol DA, Genuis SJ, Frye RE. Environmental toxicants and autism spectrum disorders: a systematic review. Transl Psychiatry. 2014;4:e360 10.1038/tp.2014.4 ; PubMed Central PMCID: PMCPMC3944636. 24518398 PMC3944636

[23] Shelton JF, Geraghty EM, Tancredi DJ, Delwiche LD, Schmidt RJ, Ritz B, et al Neurodevelopmental disorders and prenatal residential proximity to agricultural pesticides: the CHARGE study. Environ Health Perspect. 2014;122(10):1103–9. 10.1289/ehp.1307044 ; PubMed Central PMCID: PMCPMC4181917. 24954055 PMC4181917

[24] Landrigan PJ. What causes autism? Exploring the environmental contribution. Curr Opin Pediatr. 2010;22(2):219–25. 10.1097/MOP.0b013e328336eb9a . 20087185

[25] Stewart AM, Nguyen M, Wong K, Poudel MK, Kalueff AV. Developing zebrafish models of autism spectrum disorder (ASD). Prog Neuropsychopharmacol Biol Psychiatry. 2014;50:27–36. 10.1016/j.pnpbp.2013.11.014 . 24315837

[26] Kurian JR, Forbes-Lorman RM, Auger AP. Sex Difference in Mecp2 Expression During a Critical Period of Rat Brain Development. Epigenetics. 2007;2(3):173–8. 10.4161/epi.2.3.4841 17965589

[27] Silverman JL, Yang M, Lord C, Crawley JN. Behavioural phenotyping assays for mouse models of autism. Nat Rev Neurosci. 2010;11(7):490–502. 10.1038/nrn2851 ; PubMed Central PMCID: PMCPMC3087436. 20559336 PMC3087436

[28] Kalueff AV, Stewart AM, Gerlai R. Zebrafish as an emerging model for studying complex brain disorders. Trends Pharmacol Sci. 2014;35(2):63–75. 10.1016/j.tips.2013.12.002 ; PubMed Central PMCID: PMCPMC3913794. 24412421 PMC3913794

[29] Hill AJ, Teraoka H, Heideman W, Peterson RE. Zebrafish as a model vertebrate for investigating chemical toxicity. Toxicol Sci. 2005;86(1):6–19. 10.1093/toxsci/kfi110 . 15703261

[30] Tropepe V, Sive HL. Can zebrafish be used as a model to study the neurodevelopmental causes of autism? Genes, Brain and Behavior. 2003;2(5):268–81. 10.1034/j.1601-183X.2003.00038.x 14606692

[31] Bailey JM, Oliveri AN, Karbhari N, Brooks RA, De La Rocha AJ, Janardhan S, et al Persistent behavioral effects following early life exposure to retinoic acid or valproic acid in zebrafish. Neurotoxicology. 2016;52:23–33. 10.1016/j.neuro.2015.10.001 ; PubMed Central PMCID: PMCPMC4753107. 26439099 PMC4753107

[32] Kozol RA, Cukier HN, Zou B, Mayo V, De Rubeis S, Cai G, et al Two knockdown models of the autism genes SYNGAP1 and SHANK3 in zebrafish produce similar behavioral phenotypes associated with embryonic disruptions of brain morphogenesis. Hum Mol Genet. 2015;24(14):4006–23. 10.1093/hmg/ddv138 ; PubMed Central PMCID: PMCPMC4476447. 25882707 PMC4476447

[33] Maaswinkel H, Zhu L, Weng W. Assessing social engagement in heterogeneous groups of zebrafish: a new paradigm for autism-like behavioral responses. PLoS One. 2013;8(10):e75955 10.1371/journal.pone.0075955 ; PubMed Central PMCID: PMCPMC3792997. 24116082 PMC3792997

[34] Zimmermann FF, Gaspary KV, Leite CE, De Paula Cognato G, Bonan CD. Embryological exposure to valproic acid induces social interaction deficits in zebrafish (*Danio rerio*): A developmental behavior analysis. Neurotoxicol Teratol. 2015;52(Pt A):36–41. 10.1016/j.ntt.2015.10.002 . 26477937

[35] Dobin A, Davis CA, Schlesinger F, Drenkow J, Zaleski C, Jha S, et al STAR: ultrafast universal RNA-seq aligner. Bioinformatics. 2013;29(1):15–21. 10.1093/bioinformatics/bts635 23104886 PMC3530905

[36] Trapnell C, Pachter L, Salzberg SL. TopHat: discovering splice junctions with RNA-Seq. Bioinformatics. 2009;25(9):1105–11. 10.1093/bioinformatics/btp120 19289445 PMC2672628

[37] Anders S, Pyl PT, Huber W. HTSeq—a Python framework to work with high-throughput sequencing data. Bioinformatics. 2015;31(2):166–9. 10.1093/bioinformatics/btu638 25260700 PMC4287950

[38] Sun J, Nishiyama T, Shimizu K, Kadota K. TCC: an R package for comparing tag count data with robust normalization strategies. BMC Bioinformatics. 2013;14(1):219 10.1186/1471-2105-14-219 23837715 PMC3716788

[39] Sanders SJ, He X, Willsey AJ, Ercan-Sencicek AG, Samocha KE, Cicek AE, et al Insights into Autism Spectrum Disorder Genomic Architecture and Biology from 71 Risk Loci. Neuron. 2015;87(6):1215–33. Epub 2015/09/25. 10.1016/j.neuron.2015.09.016 ; PubMed Central PMCID: PMCPMC4624267 26402605 PMC4624267

[40] van Aerle R, Lange A, Moorhouse A, Paszkiewicz K, Ball K, Johnston BD, et al Molecular mechanisms of toxicity of silver nanoparticles in zebrafish embryos. Environ Sci Technol. 2013;47(14):8005–14. 10.1021/es401758d ; PubMed Central PMCID: PMCPMC3854648. 23758687 PMC3854648

[41] Jasien JM, Daimon CM, Wang R, Shapiro BK, Martin B, Maudsley S. The effects of aging on the BTBR mouse model of autism spectrum disorder. Front Aging Neurosci. 2014;6:225 10.3389/fnagi.2014.00225 ; PubMed Central PMCID: PMCPMC4150363. 25225482 PMC4150363

[42] Livak KJ, Shumittgen TD. Analysis of relative gene expression data using real-time quantitative PCR and the 2(-Delta Delta C(T)) Method. Methods. 2001; 25: 402–408. dio: 10.1006/meth.2001.1262 11846609

[43] Ku HO, Jeong SH, Kang HG, Pyo HM, Cho JH, et al Gene expression profiles and pathways in skin inflamation induced by three different sensitizers and an irritant. Toxicol Lett. 2009;190:231–237. 10.1016/j.toxlet.2009.07.022 19647056

[44] Kumar R, Samal S, Routray S, Dash R, Dixit A. Identification of oral cancer related cadidate genes by integrating protein-protein interactions, gene ontology, pathway analysis and immunohistochemistry. Scientific Reports. 2017;7:2472 10.1038/s41598-017-02522-5 28559546 PMC5449392

[45] Stone RL, Aimi J, Barshop BA, Jaeken J, Van den Berghe G, Zalkin H, et al A mutation in adenylosuccinate lyase associated with mental retardation and autistic features. Nat Genet. 1992;1:59 10.1038/ng0492-59 1302001

[46] FE A., Julie S, Catherine M, Julio A, SM I., Anne P, et al Adenylosuccinate lyase (ADSL) and infantile autism: Absence of previously reported point mutation. Am J Med Genet. 1995;60(6):554–7. 10.1002/ajmg.1320600614 8825895

[47] Jurecka A, Zikanova M, Tylki-Szymanska A, Krijt J, Bogdanska A, Gradowska W, et al Clinical, biochemical and molecular findings in seven Polish patients with adenylosuccinate lyase deficiency. Mol Genet Metab. 2008;94(4):435–42. Epub 2008/06/06. 10.1016/j.ymgme.2008.04.013 . 18524658

[48] S L. Getting to the bottom of autism spectrum and related disorders: MBD5 as a key contributor. Clinical Genetics. 2012;81(4):363–4. 10.1111/j.1399-0004.2011.01836.x 22171639

[49] Talkowski ME, Mullegama SV, Rosenfeld JA, van Bon BW, Shen Y, Repnikova EA, et al Assessment of 2q23.1 microdeletion syndrome implicates MBD5 as a single causal locus of intellectual disability, epilepsy, and autism spectrum disorder. Am J Hum Genet. 2011;89(4):551–63. Epub 2011/10/11. 10.1016/j.ajhg.2011.09.011 ; PubMed Central PMCID: PMCPMC3188839. 21981781 PMC3188839

[50] Peca J, Feliciano C, Ting JT, Wang W, Wells MF, Venkatraman TN, et al Shank3 mutant mice display autistic-like behaviours and striatal dysfunction. Nature. 2011;472(7344):437–42. 10.1038/nature09965 ; PubMed Central PMCID: PMCPMC3090611. 21423165 PMC3090611

[51] Costa-Mattioli M, Monteggia LM. mTOR complexes in neurodevelopmental and neuropsychiatric disorders. Nat Neurosci. 2013;16(11):1537–43. 10.1038/nn.3546 . 24165680

[52] Shih BB, Nirmal AJ, Headon DJ, Akbar AN, Mabbott NA et al Derivation of marker gene signatures from human skin and their use in the interpretation of the transcriptional changes associated with dermatological disorders. J Pathol. 2017;241:600–613. 10.1002/path.4864 28008606 PMC5363360

[53] Sztajnkrycer MD. Valproic acid toxicity: overview and management. J Toxicol Clin Toxicol. 2002;40:789–801. 10.1081/CLT-120014645 12475192

[54] Anitha A, Nakamura K, Thanseem I, Yamada K, Iwayama Y, Toyota T, et al Brain region-specific altered expression and association of mitochondria-related genes in autism. Mol Autism. 2012;3(1):12 10.1186/2040-2392-3-12 23116158 PMC3528421

[55] Hollis F, Kanellopoulos AK, Bagni C. Mitochondrial dysfunction in Autism Spectrum Disorder: clinical features and perspectives. Curr Opin Neurobiol. 2017;45:178–87. Epub 2017/06/20. 10.1016/j.conb.2017.05.018 . 28628841

[56] Siddiqui MF, Elwell C, Johnson MH. Mitochondrial Dysfunction in Autism Spectrum Disorders. Autism Open Access. 2016;6(5). Epub 2016/12/09. 10.4172/2165-7890.1000190 ; PubMed Central PMCID: PMCPMC5137782. 27928515 PMC5137782

[57] Schneider T, Przewlocki R. Behavioral alterations in rats prenatally exposed to valproic acid: animal model of autism. Neuropsychopharmacology. 2005;30(1):80–9. 10.1038/sj.npp.1300518 . 15238991

[58] Beker van Woudenberg A, Snel C, Rijkmans E, de Groot D, Bouma M, Hermsen S, et al Zebrafish embryotoxicity test for developmental (neuro)toxicity: Demo case of an integrated screening approach system using anti-epileptic drugs. Reprod Toxicol. 2014;49:101–16. 10.1016/j.reprotox.2014.07.082 . 25111975

