# Supplementary figures and images for: Plausibility of the zebrafish embryos/larvae as an alternative animal model for autism: A comparison study of transcriptome changes
Source: PLoS One. 2018 Sep 4;13(9):e0203543. doi: 10.1371/journal.pone.0203543 (PMC6122816; doi:10.1371/journal.pone.0203543)

**
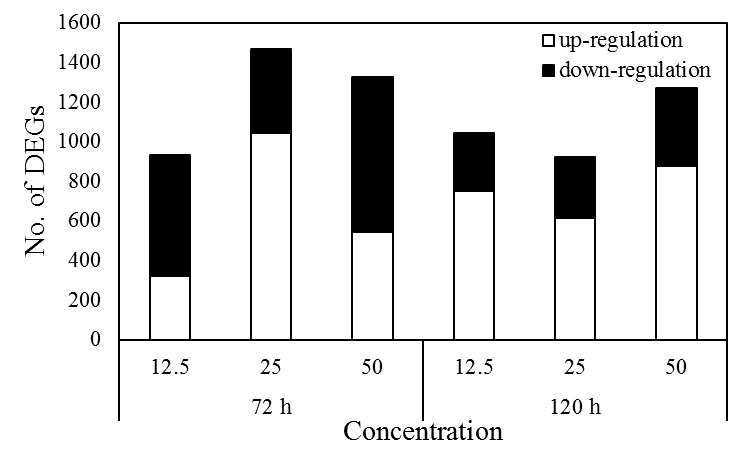
**

**S1 Fig. Differentially expressed genes (DEGs) after exposure to VPA for 72 h and 120 h.**

Supplement: S1 Fig — (DOCX) [file pone.0203543.s001.docx]
