# Supplementary material for: Plausibility of the zebrafish embryos/larvae as an alternative animal model for autism: A comparison study of transcriptome changes
Source: PLoS One. 2018 Sep 4;13(9):e0203543. doi: 10.1371/journal.pone.0203543 (PMC6122816; doi:10.1371/journal.pone.0203543)
Supplement: S2 Fig — Comparison of commonalities of (a) DEGs and (b) GOs between ASD (shown in Fig 3) and other oral and skin-related diseases. (DOCX) [file pone.0203543.s002.docx]

| (a) | **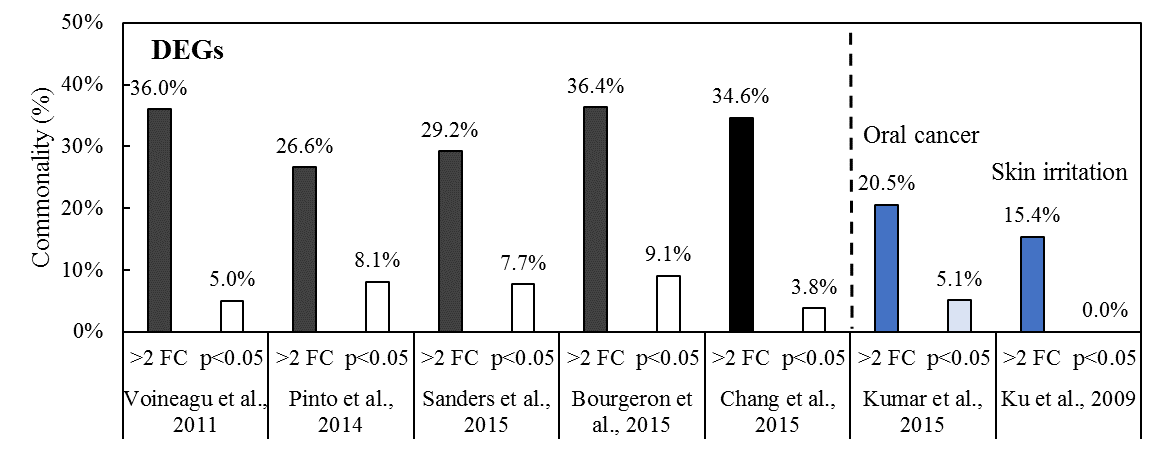** |
| --- | --- |
| (b) | **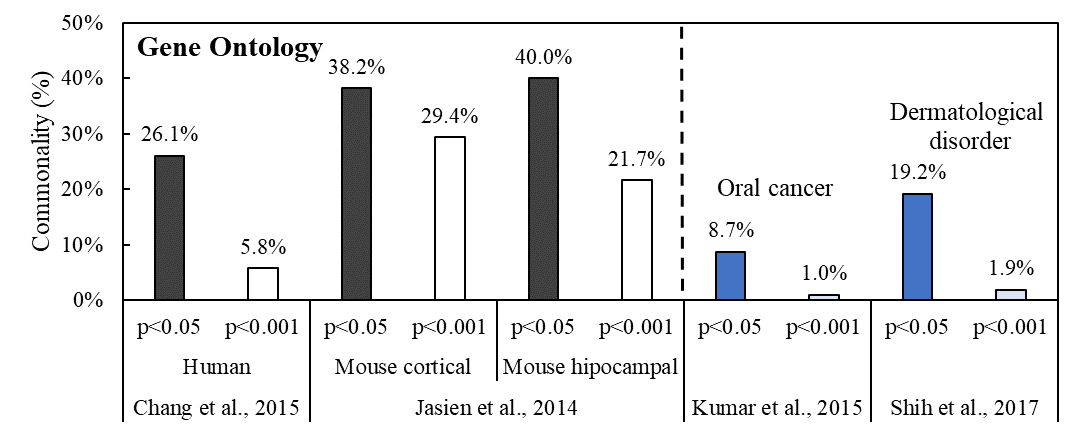** |

**S2 Fig. Comparison of commonalities in (a) DEGs and (b) GOs between ASD (shown in Fig. 3) and oral and skin related disease.**
