# Supplementary material for: Plausibility of the zebrafish embryos/larvae as an alternative animal model for autism: A comparison study of transcriptome changes
Source: PLoS One. 2018 Sep 4;13(9):e0203543. doi: 10.1371/journal.pone.0203543 (PMC6122816; doi:10.1371/journal.pone.0203543)
Supplement: S1 Table — (DOCX) [file pone.0203543.s003.docx]

**S1 Table. Primer sequences for qRT-PCR analysis used in this study**

| Gene | Primer Sequence (5’-3’) | Accession no. |
| --- | --- | --- |
| *adsl* | F: CCAGCAAAGAGATGGCCTAC  R: TGACATCATGCCTGAGCTTC | NM_199899.2 |
| *mbd5* | F: GTGTGTGTGTCGGACTGGAC  R: GCAGAGACACGACAAGACCA | XR_002459017.1 |
| *shank3a* | F: CTGTTTTACGGAGCGGACAT  R: CCCTGAAAGGCACAACATCT | LM994718.1 |
| *tsc1b* | F: AGCTGCAACACCTCCTCCTA  R: TCACCTCTGCCATCTCTGTG | NM_001282392.1 |
| *beta-actin* | F: CCAAAGCCAACAGAGAGAAGA  R: GATCGACCAGAAGCATACAGAG | BC045846.1 |

* from Ensembl database (https://asia.ensembl.org)
