# Supplementary material for: Plausibility of the zebrafish embryos/larvae as an alternative animal model for autism: A comparison study of transcriptome changes
Source: PLoS One. 2018 Sep 4;13(9):e0203543. doi: 10.1371/journal.pone.0203543 (PMC6122816; doi:10.1371/journal.pone.0203543)
Supplement: S2 Table — (DOCX) [file pone.0203543.s004.docx]

**S2 Table. Statistics for read mapping**

| **Time** | | **Group** | **Total reads** | **Cleaned reads** | **Mapped reads** | **Uniquely mapped reads** | **No. of reads properly paired genes** |
| --- | --- | --- | --- | --- | --- | --- | --- |
| 72 h | Control | | 77,940,090 | 77,258,260  (99.1%) | 62,235,761  (88.3%) | 65,931,410  (85.3%) | 64,401,080  (83.36%) |
|  | 12.5 | | 89,404,900 | 88,455,874  (98.9%) | 75,994,642  (85.9%) | 73,194,457  (82.7%) | 68,474,760  (77.41%) |
|  | 25.0 | | 90,572,046 | 89,398,276  (98.7%) | 73,854,119  (82.6%) | 70,828,913  (79.2%) | 62,560,144  (69.98%) |
|  | 50.0 | | 90,818,122 | 89,837,770  (98.9%) | 75,713,361  (84.3%) | 72,419,165  (80.6%) | 65,534,224  (72.95%) |
| 120 h | Control | | 77,643,790 | 76,586,952  (98.6%) | 63,039,673  (82.3%) | 60,577,370  (79.1%) | 53,428,706  (69.76%) |
|  | 12.5 | | 77,050,282 | 75,916,592  (98.5%) | 62,442,094  (82.3%) | 59,850,699  (78.8%) | 52,585,934  (69.27%) |
|  | 25.0 | | 78,001,026 | 77,019,964  (98.7%) | 65,622,819  (85.2%) | 62,934,742  (81.7%) | 58,754,874  (76.29%) |
|  | 50.0 | | 76,546,746 | 75,434,676  (98.5%) | 66,155,566  (87.7%) | 63,808,522  (84.6%) | 61,954,036  (82.13%) |
