# Supplementary material for: Plausibility of the zebrafish embryos/larvae as an alternative animal model for autism: A comparison study of transcriptome changes
Source: PLoS One. 2018 Sep 4;13(9):e0203543. doi: 10.1371/journal.pone.0203543 (PMC6122816; doi:10.1371/journal.pone.0203543)
Supplement: S3 Table — (DOCX) [file pone.0203543.s005.docx]

**S3 Table. Differentially expressed genes after VPA exposure in zebrafish embryo/larvae among the ASD related genes suggested by Voineagu *et al*. (2011)**

| **Gene** | **Description** | **Log_2_FC^1)^** | | | | | |
| --- | --- | --- | --- | --- | --- | --- | --- |
|  |  | **72h** | | | **120 h** | | |
|  |  | **12.5** | **25** | **50** | **12.5** | **25** | **50** |
| ***adsl*** | **adenylosuccinate lyase** | -0.49 | **-1.58*** | -1.12 | -0.13 | 0.74 | **1.46** |
| *arx* | aristaless related homeobox a | -0.45 | **-1.37** | -0.93 | -0.04 | 0.61 | **1.74** |
| *astn2* | astrotactin-2 | **-1.71** | **-3.94** | **-3.16** | 0.11 | **1.95** | **2.47** |
| *avpr1a* | arginine vasopressin receptor 1Ab | -0.52 | -0.92 | **-1.10** | -0.21 | -0.21 | 0.77 |
| *cap2* | Ca^++^-dependent secretion activator 2 | -0.72 | **-1.34** | **-1.46** | -0.34 | -0.10 | 0.49 |
| *cd83* | CD83 molecule | 0.39 | -0.47 | **1.15** | 0.33 | -0.04 | **-1.12** |
| *cdh18* | cadherin-18 | 0.56 | -0.11 | -0.69 | 0.36 | -0.06 | **1.04** |
| *cdkl5* | cyclin-dependent kinase-like 5 | **-1.88** | NA | **-3.56** | NA | NA | NA |
| *cntn3* | contactin-3 | 0.24 | 0.66 | **1.11** | -0.41 | -0.61 | **-1.20** |
| *dhcr7* | 7-dehydrocholesterol reductase | -0.23 | -0.52 | -0.58 | 0.31 | 0.46 | **1.23** |
| *dmd* | dystrophin | -0.39 | **-1.13** | -0.73 | -0.29 | 0.19 | 0.56 |
| *dpyd* | dihydropyrimidine dehydrogenase b | -0.66 | -0.90 | **-1.39** | 0.17 | 0.45 | **1.38** |
| *eif4e* | eukaryotic translation initiation factor 4E family member 1c | -0.19 | -0.41 | -0.62 | 0.39 | 0.83 | **1.22** |
| *fhit* | si:ch211-63i20.3 | NA | NA | NA | -1.97 | -1.18 | NA |
| *gabrb1* | gamma-aminobutyric acid (GABA) A receptor, beta 1 | 0.14 | -0.53 | **-1.03** | 0.12 | -0.20 | -0.36 |
| *grid1* | glutamate receptor, ionotropic, delta 1a | -0.21 | **-1.11** | -0.95 | -0.15 | -0.15 | 0.66 |
| *grm8* | metabotropic glutamate receptor 8 | -0.53 | **-1.28** | -0.80 | 0.24 | 0.61 | **1.12** |
| *grpr* | gastrin-releasing peptide receptor | -0.15 | -0.88 | **-1.38** | -0.58 | -0.30 | -0.33 |
| *htr3a* | 5-hydroxytryptamine receptor 3A | -0.93 | **-1.23** | -0.41 | **1.43** | **1.51** | -0.78 |
| *inpp1* | si:ch211-160o17.2 | -0.32 | -0.96 | -0.31 | 0.13 | 0.84 | **1.66** |
| *kcnma1* | calcium-activated potassium channel subunit alpha-1 | **-2.55** | NA | **-2.46** | NA | NA | NA |
| *lamb1* | laminin subunit beta-1 | **-1.51** | **-1.64** | -0.70 | -0.96 | -0.75 | -0.72 |
| *mdga2* | MAM domain-containing glycosyl  -phosphatidylinositol anchor protein 2 | NA | NA | **-2.09** | NA | NA | NA |
| *mtf1* | metal regulatory transcription factor 1 | -0.39 | **-2.18** | **-1.44** | 0.65 | **1.45** | **2.75** |
| *nbea* | neurobeachin a | -0.25 | -0.78 | -0.54 | -0.30 | 0.09 | **1.10** |
| *nsd1* | nuclear receptor binding SET domain protein 1b | 0.04 | -0.56 | -0.06 | -0.68 | **-1.06** | -0.61 |
| *oxtr* | oxytocin receptor | -0.25 | **-1.12** | -0.26 | -0.14 | -0.23 | -0.25 |
| *pappa2* | pappalysin 2 | -0.35 | **-1.10** | **-1.03** | -0.09 | 0.25 | 0.58 |
| *park2* | parkin RBR E3 ubiquitin protein ligase | **-3.21** | **-3.63** | **-3.97** | **1.66** | 0.22 | **1.07** |
| *pcdh10* | protocadherin 10b | -0.23 | -0.61 | -0.46 | -0.41 | -0.23 | **1.15** |
| ***pde9a*** | **high affinity cGMP-specific 3',5'-cyclic phosphodiesterase 9A** | -0.12 | **-1.04** | **-1.34** | **1.05*** | **1.23*** | 0.71 |
| *pip5k1b* | phosphatidylinositol 4-phosphate 5-kinase type-1 beta | -0.25 | **-1.85** | **-1.42** | -0.30 | 0.05 | 0.08 |
| *pknox1* | homeobox protein PKNOX1 | 0.49 | 0.49 | 0.35 | -0.20 | -0.32 | **-1.05** |
| *rnf8* | ring finger protein 8, E3 ubiquitin protein ligase | -0.55 | -0.60 | -0.54 | 0.06 | 0.40 | **1.28** |
| ***rpl10*** | **ribosomal protein L10** | -0.76 | **-2.21*** | **-1.75** | -0.13 | 0.46 | **1.85*** |
| *shank1* | SH3 and multiple ankyrin repeat domains protein 1 | 0.34 | **-1.59** | **-1.54** | 0.53 | -0.39 | -0.06 |
| *shank2* | SH3 and multiple ankyrin repeat domains protein 2 | -0.24 | -0.64 | -0.75 | **1.44** | 0.68 | **1.17** |
| ***shank3*** | **SH3 and multiple ankyrin repeat domains 3a** | 0.82 | 0.59 | 0.17 | 0.87* | 0.39 | 0.73 |
| *slc25a12* | solute carrier family 25 (aspartate/glutamate carrier), member 12 | -0.14 | -0.53 | -0.82 | 0.34 | 0.57 | **1.15** |
| *slc4a10* | solute carrier family 4, sodium bicarbonate transporter, member 10b | 0.10 | **-1.10** | **-1.33** | 0.07 | 0.04 | 0.77 |
| *slc6a4* | solute carrier family 6 (neurotransmitter transporter), member 4b | 0.14 | **-1.57** | **-1.67** | -0.18 | -0.02 | -0.28 |
| *srpk2* | SRSF protein kinase 2 | -0.11 | -0.87 | -0.68 | -0.14 | 0.04 | **1.45** |
| *st7* | suppression of tumorigenicity 7 | -0.94 | **-1.95** | **-1.66** | -0.49 | 0.19 | **1.63** |
| ***suclg2*** | **succinate-CoA ligase, GDP-forming, beta subunit** | -0.61 | **-1.43** | **-2.14*** | 0.25 | 0.56 | **1.35** |
| *tbx1* | T-box transcription factor TBX1 | -0.70 | 0.12 | -0.01 | **4.17** | -0.24 | **4.01** |
| ***tdo2*** | **tryptophan 2,3-dioxygenase a** | 0.25 | 0.07 | **-1.82*** | 0.34 | 0.33 | 0.18 |
| ***tsc1*** | **tuberous sclerosis 1b** | -0.05 | -0.20 | -0.03 | -0.67* | -0.69 | -0.61 |
| *tsc2* | tuberous sclerosis 2 | -0.57 | -0.83 | -0.77 | -0.18 | -0.02 | **1.18** |
| *vps13b* | vacuolar protein sorting-associated protein 13B | -0.44 | NA | NA | 0.00 | -0.40 | **6.36** |
| *wnt2* | wingless-type MMTV integration site family member 2 | **-1.07** | **-1.92** | **-1.47** | -0.42 | 0.47 | **2.85** |

1) The value of | log_2_FC | > 1 is marked in bold. Asterisk (*) indicates a statistical significance (*P* < 0.05). NA: not available (data under FPKM cut-off value (0.1)).
