# Supplementary material for: Plausibility of the zebrafish embryos/larvae as an alternative animal model for autism: A comparison study of transcriptome changes
Source: PLoS One. 2018 Sep 4;13(9):e0203543. doi: 10.1371/journal.pone.0203543 (PMC6122816; doi:10.1371/journal.pone.0203543)
Supplement: S4 Table — (DOCX) [file pone.0203543.s006.docx]

**S4 Table. Differentially expressed genes after VPA exposure in zebrafish embryo/larvae among the ASD related genes suggested by Pinto *et al*. (2014)**

| **Gene** | **Description** | **Log_2_FC^1)^** | | | | | |
| --- | --- | --- | --- | --- | --- | --- | --- |
|  |  | **72h** | | | **120 h** | | |
|  |  | **12.5** | **25** | **50** | **12.5** | **25** | **50** |
| ***adsl*** | **adenylosuccinate lyase** | -0.49 | **-1.58*** | **-1.12** | -0.13 | 0.74 | **1.46** |
| *aldh5a1* | aldehyde dehydrogenase 5 family, member A1 (succinate-semialdehyde dehydrogenase) | -0.42 | -0.76 | **-1.13** | -0.13 | -0.05 | 0.67 |
| *arx* | aristaless related homeobox a | -0.45 | **-1.37** | -0.93 | -0.04 | 0.61 | **1.74** |
| *bbs10* | Bardet-Biedl syndrome 10 | **-1.09** | -0.51 | -0.63 | -0.09 | 0.44 | 0.78 |
| *btd* | biotinidase | **-1.05** | **-1.75** | **-2.17** | -0.18 | -0.58 | 0.81 |
| *cask* | peripheral plasma membrane protein CASK | -0.77 | **-1.42** | **-1.00** | 0.29 | 0.29 | **2.29** |
| *cdkl5* | cyclin-dependent kinase-like 5 | -1.88 | NA | -3.56 | NA | 0.00 | NA |
| ***dcx*** | **dicarbonyl/L-xylulose reductase** | **-1.66*** | -1.72 | **-2.07*** | -0.66 | -0.39 | **1.65** |
| *dhcr7* | 7-dehydrocholesterol reductase | -0.23 | -0.52 | -0.58 | 0.31 | 0.46 | **1.23** |
| *dmd* | dystrophin | -0.39 | **-1.13** | -0.73 | -0.29 | 0.19 | 0.56 |
| *dpyd* | dihydropyrimidine dehydrogenase b | -0.66 | -0.9 | **-1.39** | 0.17 | 0.45 | **1.38** |
| *dyrk1a* | dual-specificity tyrosine-(Y)-phosphorylation regulated kinase 1A, a | -0.50 | **-1.13** | **-1.34** | 0.57 | 0.24 | **2.03** |
| *foxg1* | forkhead box G1a | -0.47 | 0.00 | -0.78 | 0.28 | 0.62 | **2.52** |
| ***gamt*** | **guanidinoacetate N-methyltransferase** | -0.60 | **-1.24** | **-1.43*** | 0.06 | 0.59 | **1.35** |
| ***gatm*** | **glycine amidinotransferase**  **(L-arginine:glycine amidinotransferase)** | **-1.67*** | **-2.6** | **-3.44*** | **1.05** | **1.16** | **5.08*** |
| *gns* | glucosamine (N-acetyl)-6-sulfatase (Sanfilippo disease IIID), b | 0.02 | -0.98 | -0.93 | **2.93** | **3.48** | NA |
| *gria3* | glutamate receptor, ionotropic, AMPA 3a | 0.08 | -0.94 | **-1.14** | 0.03 | -0.15 | -0.22 |
| ***hsd17b10*** | **hydroxysteroid (17-beta) dehydrogenase 10** | **-0.88*** | **-1.43** | **-1.42*** | -0.07 | 0.16 | **1.41** |
| *iqsec2* | IQ motif and Sec7 domain 2 | -0.01 | -0.32 | **-1.01** | -0.19 | -0.49 | -0.63 |
| ***kcnj11*** | **potassium inwardly-rectifying channel, subfamily J, member 11** | -0.71 | **-1.99*** | **-2.12*** | 0.44 | 0.87 | **2.38*** |
| ***mbd5*** | **methyl-cpg binding domain protein 5** | **-1.38*** | **-3.34** | **-3.39** | 0.19 | **-1.34** | **4.83** |
| *mef2c* | myocyte enhancer factor 2ca | -0.22 | **-1.06** | 0.36 | 0.27 | 0.53 | -0.10 |
| *nsd1* | nuclear receptor binding SET domain protein 1b | 0.04 | -0.56 | -0.06 | -0.68 | **-1.06** | -0.61 |
| ***rpe65*** | **retinal pigment epithelium-specific protein 65a** | -0.33 | -0.77 | **-1.56*** | -0.70 | **1.02*** | **1.01** |
| *scn1a* | sodium channel, voltage-gated, type I, alpha | 0.02 | **-1.03** | -0.16 | 0.30 | 0.48 | 0.49 |
| *scn8a* | sodium channel protein type 8 subunit alpha | **-1.43** | **-2.60** | NA | NA | NA | NA |
| *shank2* | SH3 and multiple ankyrin repeat domains protein 2 | -0.24 | -0.64 | -0.75 | **1.44** | 0.68 | **1.17** |
| ***shank3*** | **SH3 and multiple ankyrin repeat domains protein 3a** | 0.82 | 0.59 | 0.17 | **0.87*** | 0.39 | 0.73 |
| *smad4* | expressed sequence CR929477 | -0.68 | -0.39 | -0.2 | 0.08 | 0.07 | **1.11** |
| *tcf4* | transcription factor 4 | 0.05 | **-2.18** | -0.62 | **-1.30** | -0.05 | 0.75 |
| ***tsc1*** | **tuberous sclerosis 1a** | -0.05 | -0.20 | -0.03 | **-0.67*** | -0.69 | -0.61 |
| *tsc2* | tuberous sclerosis 2 | -0.57 | -0.83 | -0.77 | -0.18 | -0.02 | **1.18** |
| *vps13b* | vacuolar protein sorting-associated protein 13B | -0.44 | NA | NA | 0.00 | -0.40 | **6.36** |

1) The value of | log_2_FC | > 1 is marked in bold. Asterisk (*) indicates a statistical significance (*P* < 0.05). NA: not available (data under FPKM cut-off value (0.1)).
