# Supplementary material for: Plausibility of the zebrafish embryos/larvae as an alternative animal model for autism: A comparison study of transcriptome changes
Source: PLoS One. 2018 Sep 4;13(9):e0203543. doi: 10.1371/journal.pone.0203543 (PMC6122816; doi:10.1371/journal.pone.0203543)
Supplement: S5 Table — (DOCX) [file pone.0203543.s007.docx]

**S5 Table. Differentially expressed genes after VPA exposure in zebrafish embryo/larvae among the ASD related genes suggested by Sanders *et al*. (2015)**

| **Gene** | **Description** | **Log_2_FC^1)^** | | | | | |
| --- | --- | --- | --- | --- | --- | --- | --- |
|  |  | **72h** | | | **120 h** | | |
|  |  | **12.5** | **25** | **50** | **12.5** | **25** | **50** |
| *ank2* | ankyrin 2b | 0.36 | -0.03 | **1.03** | 0.32 | -0.12 | **-1.2** |
| *capn12* | calcium-activate neutral proteinase 12 | -0.14 | -0.96 | -0.81 | 0.37 | **1.22*** | **1.47** |
| *cttnbp2* | cortactin binding protein 2 | -0.37 | **-1.09** | **-1.22** | -0.37 | 0.03 | 0.72 |
| *dscam* | Down syndrome cell adhesion molecule a | -0.46 | -0.88 | **-1.17** | -0.25 | -0.33 | **1.16** |
| *dyrk1a* | dual-specificity tyrosine-(Y)-phosphorylation regulated kinase 1A, a | -0.5 | **-1.13** | **-1.34** | 0.57 | 0.24 | **2.03** |
| *ilf2* | interleukin enhancer binding factor 2 | -0.39 | -0.74 | -0.7 | -0.17 | 0.57 | **1.43** |
| *katnal2* | katanin p60 subunit A-like 2 | -0.9 | -0.73 | **-1.82** | 0.17 | -0.17 | -0.64 |
| *kdm5b* | lysine (K)-specific demethylase 5Ba | -0.21 | **-1.03** | -0.5 | -0.16 | 0.32 | 0.79 |
| *kmt2e* | lysine (K)-specific methyltransferase 2E | -0.55 | **-1.07** | -0.74 | -0.18 | -0.06 | 0.7 |
| *mbd5* | methyl-CpG binding domain protein 5 | **-1.38*** | **-3.34** | **-3.39** | 0.19 | **-1.34** | **4.83*** |
| *mfrp* | Membrane frizzled-related protein | **-1.25** | **-1.59** | -0.5 | **-1.54** | **1.1** | -0.33 |
| *ninl* | Ninein-like protein | -0.45 | **-1.48** | **-1.46** | 0.2 | 0.1 | 0.76 |
| *ptk7* | Inactive tyrosine-protein kinase 7 | **-1.02** | **-1.87** | **-4.17** | -0.01 | 0.37 | **4.37** |
| *scn2a* | Sodium channel protein type 2 subunit alpha | 0.77 | **-1.99** | -0.7 | **1.34** | **1.43** | **2.28** |
| *shank2* | SH3 and multiple ankyrin repeat domains protein 2 | -0.24 | -0.64 | -0.75 | **1.44** | 0.68 | **1.17** |
| *shank3* | SH3 and multiple ankyrin repeat domains 3a | 0.82 | 0.59 | 0.17 | 0.87* | 0.39 | 0.73 |
| *slc6a1* | solute carrier family 6 (neurotransmitter transporter, GABA), member 1, like | -0.06 | -0.08 | **-3.58*** | -0.73 | **-1.16** | **-1.86** |
| *syngap1* | synaptic Ras GTPase activating protein 1a | **1.15** | 0.77 | 0.99 | 0.09 | -0.18 | **-4.3** |
| *trip12* | thyroid hormone receptor interactor 12 | **-1.33** | **-1.94** | **-1.64** | 0.04 | 0.28 | **2.57** |

1) The value of | log_2_FC | > 1 is marked in bold. Asterisk (*) indicates a statistical significance (*P* < 0.05).
