# Supplementary material for: Plausibility of the zebrafish embryos/larvae as an alternative animal model for autism: A comparison study of transcriptome changes
Source: PLoS One. 2018 Sep 4;13(9):e0203543. doi: 10.1371/journal.pone.0203543 (PMC6122816; doi:10.1371/journal.pone.0203543)
Supplement: S6 Table — (DOCX) [file pone.0203543.s008.docx]

**S6 Table. Differentially expressed genes after VPA exposure in zebrafish embryo/larvae among the ASD related genes suggested by Bourgeron *et al*. (2014)**

| **Gene** | **Description** | **Log_2_FC^1)^** | | | | | |
| --- | --- | --- | --- | --- | --- | --- | --- |
|  |  | **72 h** | | | **120 h** | | |
|  |  | **12.5** | **25** | **50** | **12.5** | **25** | **50** |
| *ank2* | ankyrin 2b, neuronal | 0.36 | -0.03 | **1.03** | 0.32 | -0.12 | -1.20 |
| *cask* | peripheral plasma membrane protein CASK | -0.77 | **-1.42** | **-1.00** | 0.29 | 0.29 | **2.29** |
| *ctnnb1* | catenin (cadherin-associated protein), beta 1 | -0.72 | -0.50 | **-1.13** | -0.06 | -0.25 | **1.18** |
| *dyrk1a* | dual-specificity tyrosine-(Y)-phosphorylation regulated kinase 1A, a | -0.38 | -0.88 | -0.94 | 0.24 | 0.61 | **1.34** |
| *mef2c* | myocyte enhancer factor 2ca | -0.22 | **-1.06** | 0.36 | 0.27 | 0.53 | -0.10 |
| ***nrxn2*** | **neurexin-2** | **-1.81*** | NA | **-5.82** | NA | NA | NA |
| *scn1a* | sodium channel, voltage-gated, type I, alpha | 0.02 | **-1.03** | -0.16 | 0.30 | 0.48 | 0.49 |
| *shank1* | SH3 and multiple ankyrin repeat domains protein 1 | 0.34 | **-1.59** | **-1.54** | 0.53 | -0.39 | -0.06 |
| *shank2* | SH3 and multiple ankyrin repeat domains protein 2 | -0.24 | -0.64 | -0.75 | **1.44** | 0.68 | **1.17** |
| ***shank3*** | **SH3 and multiple ankyrin repeat domains 3a** | 0.82 | 0.59 | 0.17 | 0.87* | 0.39 | 0.73 |
| ***tsc1*** | **tuberous sclerosis 1b** | -0.05 | -0.20 | -0.03 | -0.67* | -0.69 | -0.61 |
| *tsc2* | tuberous sclerosis 2 | -0.57 | -0.83 | -0.77 | -0.18 | -0.02 | **1.18** |

1) The value of | log_2_FC | > 1 is marked in bold. Asterisk (*) indicates a statistical significance (*P* < 0.05). NA: not available (data under FPKM cut-off value (0.1)).
