# Supplementary material for: Plausibility of the zebrafish embryos/larvae as an alternative animal model for autism: A comparison study of transcriptome changes
Source: PLoS One. 2018 Sep 4;13(9):e0203543. doi: 10.1371/journal.pone.0203543 (PMC6122816; doi:10.1371/journal.pone.0203543)
Supplement: S7 Table — (DOCX) [file pone.0203543.s009.docx]

**S7 Table. Differentially expressed genes after VPA exposure in zebrafish embryo/larvae among the ASD related genes suggested by Chang *et al*. (2014)**

| **Gene** | **Description** | **Log_2_FC^1)^** | | | | | |
| --- | --- | --- | --- | --- | --- | --- | --- |
|  |  | **72h** | | | **120 h** | | |
|  |  | **12.5** | **25** | **50** | **12.5** | **25** | **50** |
| *a2m* | alpha-2-macroglobulin | -0.51 | **-1.65** | **-5.61** | 0.56 | 0.43 | -0.34 |
| *abi2a* | abl-interactor 2a | -0.45 | **-1.13** | -0.94 | -0.08 | 0.18 | 0.93 |
| ***actn4*** | **alpha-actinin-4** | -0.4 | **-1.16** | -0.71 | 0.16 | **0.93*** | **1.21** |
| *ago1* | protein argonaute-1 | -0.69 | -0.51 | -0.43 | 0.41 | 0.18 | **1.08** |
| *apaf1* | apoptotic peptidase activating factor 1 | -0.38 | -0.84 | -0.60 | 0.18 | 0.47 | **1.05** |
| *cacna1ea* | calcium channel, voltage-dependent, R type, alpha 1E subunit a | 0.41 | **-1.01** | -0.44 | -0.33 | -0.09 | -0.78 |
| *cacna1sa* | calcium channel, voltage-dependent, L type, alpha 1S subunit, a | -0.30 | -0.86 | -0.33 | 0.10 | 0.53 | **1.03** |
| *cask* | peripheral plasma membrane protein CASK | -0.77 | **-1.42** | **-1.00** | 0.29 | 0.29 | **2.29** |
| *cit* | citron rho-interacting serine/threonine kinase | **1.42** | **3.27** | **2.68** | **3.74** | **3.77** | **2.77** |
| *clpb* | ClpB homolog, mitochondrial AAA ATPase chaperonin | 0.57 | **1.06** | 0.68 | 0.35 | -0.42 | -0.63 |
| *ctnnb1* | catenin (cadherin-associated protein), beta 1 | -0.72 | -0.50 | **-1.13** | -0.06 | -0.25 | **1.18** |
| *dcc* | netrin receptor DCC | -0.45 | **-3.48** | **-1.65** | **2.43** | **2.19** | **4.48** |
| ***ddb1*** | **damage-specific DNA binding protein 1** | **-1.20*** | **-2.42*** | **-2.92*** | 0.04 | -0.27 | **3.55*** |
| *ddr2b* | discoidin domain receptor tyrosine kinase 2b | -0.30 | **-1.04** | -0.75 | -0.23 | 0.38 | **1.41** |
| *dlg4* | disks large homolog 4 | -0.25 | -0.49 | -0.57 | 0.40 | 0.42 | **1.45** |
| *dst* | dystonin | 0.21 | 0.09 | **1.15** | 0.37 | 0.19 | -0.88 |
| *dyrk1aa* | dual-specificity tyrosine-(Y)-  phosphorylation regulated kinase 1A, a | -0.50 | **-1.13** | **-1.34** | 0.57 | 0.24 | **2.03** |
| *egfr* | epidermal growth factor receptor | -0.20 | -0.98 | **-1.68** | **1.95** | **2.25** | **1.79** |
| ***eif4a1a*** | **eukaryotic translation initiation factor 4A1A** | -0.33 | -0.29 | -0.65 | 0.71 | **0.94*** | **1.32** |
| ***fanca*** | **fanconi anemia group A protein** | -0.94 | **-1.43** | **-1.92** | 0.34 | **1.20** | **3.34*** |
| *flnaa* | filamin A, alpha (actin binding protein 280) | -0.69 | **-1.31** | -0.71 | 0.36 | 0.56 | 0.99 |
| *grm5* | glutamate receptor, metabotropic 5a | -0.03 | -0.39 | **-1.41** | 0.19 | -0.37 | 0.20 |
| *hrh2* | histamine receptor H2b | **-1.25** | 0.47 | **-1.32** | 0.16 | -0.54 | -0.26 |
| *itpr1a* | inositol 1,4,5-trisphosphate receptor, type 1a | -0.3 | -0.31 | 0.58 | -0.06 | 0.42 | **1.06** |
| *l3mbtl1* | lethal(3)malignant brain tumor-like protein 1 | **1.59** | **2.83** | **2.88** | 0.66 | -0.23 | **-2.64** |
| *lrp1aa* | low density lipoprotein receptor-related protein 1Aa | 0.09 | -0.87 | -0.58 | 0.22 | 0.86 | **1.05** |
| *lrp2a* | low density lipoprotein receptor-related protein 2a | -0.59 | -0.88 | -0.14 | -0.36 | 0.12 | **1.01** |
| *lrp2b* | low density lipoprotein receptor-related protein 2b | 0.67 | **-1.85** | **-4.54** | -0.15 | 0.04 | -0.30 |
| *lrp2bp* | LRP2 binding protein | 0.15 | -0.20 | 0.27 | **-1.03** | **-1.13** | -0.90 |
| *mst1rb* | macrophage stimulating 1 receptor b | 0.02 | -0.94 | 0.15 | **1.08** | **1.16** | **1.54** |
| ***myh11a*** | **myosin, heavy chain 11a, smooth muscle** | -0.08 | -0.91 | **-1.75*** | -0.07 | 0.25 | 0.14 |
| *myh11b* | myosin, heavy chain 11b, smooth muscle | 0.42 | 0.67 | 0.18 | 0.14 | -0.18 | **-1.35** |
| *nolc1* | nucleolar and coiled-body phosphoprotein 1 | -0.7 | **-1.32** | -0.99 | -0.02 | 0.43 | **1.22** |
| *oprl1* | opiate receptor-like 1 | -0.5 | **-1.05** | **-1.47** | -0.05 | -0.10 | 0.81 |
| *pdgfd* | platelet derived growth factor D | -0.54 | -0.35 | -0.25 | -0.10 | 0.28 | **1.34** |
| ***pfkm*** | **6-phosphofructokinase, muscle type** | 0.20 | -0.50 | -0.38 | 0.69 | **0.96*** | **1.63** |
| *ppm1db* | protein phosphatase, Mg2+/Mn2+ dependent, 1Db | -0.27 | -0.68 | -0.79 | -0.01 | 0.23 | **1.19** |
| *psen1* | presenilin 1 | -0.41 | -0.29 | -0.33 | 0.15 | 0.32 | **1.42** |
| *ptk7* | inactive tyrosine-protein kinase 7 | -0.72 | **-1.66** | **-2.21** | **1.44** | **1.52** | **4.10** |
| *ptprk* | protein tyrosine phosphatase, receptor type, K | -0.20 | -0.32 | -0.45 | 0.10 | -0.03 | **1.23** |
| ***ptprm*** | **receptor-type tyrosine-protein phosphatase mu** | **-1.20** | **-inf*** | **-3.99** | -0.01 | 0.58 | **5.87** |
| *rims1a* | regulating synaptic membrane exocytosis 1a | -0.30 | **-1.08** | **-1.14** | -0.27 | -0.24 | **1.00** |
| *ros1* | proto-oncogene tyrosine-protein kinase ROS | 0.31 | -0.24 | **-1.03** | 0.00 | 0.01 | -0.28 |
| *sdha* | succinate dehydrogenase complex, subunit A, flavoprotein (Fp) | -0.49 | **-1.42** | **-1.25** | -0.17 | 0.40 | **1.16** |
| *shank2* | SH3 and multiple ankyrin repeat domains 2 | 0.13 | -0.80 | **-1.10** | -0.18 | 0.07 | 0.38 |
| *smad2* | SMAD family member 2 | -0.33 | -0.65 | -0.54 | -0.18 | 0.49 | **1.05** |
| *stk11* | serine/threonine kinase 11 | -0.32 | -0.75 | -0.44 | -0.12 | 0.29 | **1.22** |
| *stk19* | serine/threonine kinase 19 | -0.70 | -0.14 | -0.79 | **-1.06** | **-1.64** | **-1.23** |
| *syne1b* | spectrin repeat containing, nuclear envelope 1b | -0.45 | -0.82 | -0.52 | -0.13 | -0.02 | **1.43** |
| *synrg* | synergin, gamma | -0.43 | **-1.21** | -0.78 | 0.00 | 0.52 | **1.29** |
| *tbl1xr1a* | transducin (beta)-like 1 X-linked receptor 1a | -0.28 | **-1.03** | -0.62 | -0.17 | 0.29 | 0.72 |
| *tcf7l1b* | transcription factor 7-like 1b  (T-cell specific, HMG-box) | -0.33 | **-1.06** | -0.39 | -0.14 | 0.61 | 0.85 |
| *tnk1bp1* | tankyrase 1 binding protein 1 | -0.06 | 0.55 | -0.01 | -0.19 | -0.7 | **-1.17** |
| *tsc2* | tuberous sclerosis 2 | -0.57 | -0.83 | -0.77 | -0.18 | -0.02 | **1.18** |
| *wnk2* | serine/threonine-protein kinase WNK2 | -0.79 | **-3.01** | **-1.67** | -0.87 | -0.11 | **2.37** |

1) The value of | log_2_FC | > 1 is marked in bold. Asterisk (*) indicates a statistical significance (*P* < 0.05). inf: infinite.
