# Supplementary material for: Plausibility of the zebrafish embryos/larvae as an alternative animal model for autism: A comparison study of transcriptome changes
Source: PLoS One. 2018 Sep 4;13(9):e0203543. doi: 10.1371/journal.pone.0203543 (PMC6122816; doi:10.1371/journal.pone.0203543)
Supplement: S8 Table — (DOCX) [file pone.0203543.s010.docx]

**S8 Table. Significantly affected GO after VPA exposure in zebrafish embryo/larvae among the ASD related GO suggested by Chang *et al*. (2014)**

| **Pathway** | **Ontology** | **Term** | **GO ID** | ***p*-value (at 72 h)** | | | ***p*-value (at 120 h)** | | | |
| --- | --- | --- | --- | --- | --- | --- | --- | --- | --- | --- |
|  |  |  |  | **12.5** | **25** | **50** | | **12.5** | **25** | **50** |
|  | BP | **cellular ion homeostasis** | GO:0006873 | - | - | - | | **<0.001** | - | - |
|  | MF | calcium channel activity | GO:0005262 | 0.894 | 0.648 | 0.030 | | 0.709 | 1.000 | 0.322 |
| Channel activity | CC | **i band** | GO:0031674 | 0.059 | 0.131 | 0.113 | | 0.572 | 0.032 | **<0.001** |
|  | MF | **metal ion transmembrane transporter activity** | GO:0046873 | 0.982 | 0.245 | **<0.001** | | 0.176 | 0.748 | 0.184 |
|  | BP | regulation of adenylate cyclase activity | GO:0045761 | 0.387 | 1.000 | 0.025 | | 1.000 | 1.000 | 0.405 |
|  | CC | sarcolemma | GO:0042383 | 0.097 | 0.027 | 0.509 | | 0.428 | 0.348 | 0.012 |
|  | MF | ATPase activity | GO:0016887 | 0.014 | 0.208 | 0.058 | | 0.703 | 0.178 | 0.029 |
|  | MF | ATPase activity, coupled | GO:0042623 | 0.066 | 0.135 | 0.009 | | 0.544 | 0.302 | 0.069 |
|  | MF | ATP-dependent helicase activity | GO:0008026 | 0.004 | 0.048 | 0.461 | | 0.140 | 0.193 | 0.006 |
|  | BP | cellular response to dna damage stimulus | GO:0006974 | 0.003 | 0.242 | 0.827 | | 0.866 | 0.985 | 0.541 |
| Chromatin | MF | **DNA helicase activity** | GO:0003678 | **<0.001** | 0.286 | 0.100 | | 0.218 | 0.486 | 0.032 |
| modification | BP | DNA repair | GO:0006281 | 0.004 | 0.558 | 0.904 | | 0.813 | 0.947 | 0.577 |
| /regulation | MF | **helicase activity** | GO:0004386 | **<0.001** | 0.029 | 0.146 | | 0.310 | 0.359 | **<0.001** |
|  | BP | **mRNA metabolic process** | GO:0016071 | **<0.001** | 0.767 | 0.856 | | 0.665 | 0.978 | 0.159 |
|  | BP | **mRNA processing** | GO:0006397 | **<0.001** | 0.665 | 0.841 | | 0.679 | 0.956 | 0.138 |
|  | BP | mRNA splicing, via spliceosome | GO:0000398 | 0.005 | 0.579 | 0.576 | | 0.658 | 0.949 | 0.066 |
|  | BP | regulation of gene expression, epigenetic | GO:0040029 | 0.035 | 0.847 | 0.837 | | 0.658 | 0.855 | 0.135 |
|  | BP | RNA splicing | GO:0008380 | 0.014 | 0.746 | 0.803 | | 0.827 | 0.980 | 0.196 |
|  | BP | RNA splicing, via transesterification reactions | GO:0000375 | 0.006 | 0.582 | 0.586 | | 0.666 | 0.950 | 0.068 |
|  | MF | actin binding | GO:0003779 | 0.439 | 0.387 | 0.005 | | 0.507 | 0.410 | 0.032 |
|  | CC | **actin cytoskeleton** | GO:0015629 | 0.024 | 0.146 | **<0.001** | | 0.469 | 0.457 | 0.028 |
|  | BP | actin cytoskeleton organization | GO:0030036 | 0.125 | 0.013 | 0.026 | | 0.963 | 0.990 | 0.004 |
| Neuronal | MF | actin filament binding | GO:0051015 | 0.812 | 1.000 | 0.006 | | 1.000 | 1.000 | 0.418 |
| signaling | CC | **adherens junction** | GO:0005912 | 0.009 | 0.034 | 0.015 | | 0.481 | 0.398 | **<0.001** |
|  | CC | **anchoring junction** | GO:0070161 | - | - | - | | - | - | **<0.001** |
|  | CC | axon | GO:0030424 | 0.601 | 0.802 | 0.276 | | 0.543 | 0.467 | 0.002 |
|  | BP | blood vessel development | GO:0001568 | 0.763 | 0.019 | 0.872 | | 0.973 | 0.669 | 0.183 |
|  | MF | calmodulin binding | GO:0005516 | 1.000 | 0.434 | 0.253 | | 0.013 | 0.657 | 0.680 |
|  | MF | cell adhesion molecule binding | GO:0050839 | 0.688 | 0.041 | 0.784 | | 1.000 | 0.597 | 0.116 |
|  | CC | **cell leading edge** | GO:0031252 | 0.668 | 0.060 | **<0.001** | | 0.864 | 0.539 | **<0.001** |
|  | BP | cell migration | GO:0016477 | 0.264 | 0.112 | 0.769 | | 0.822 | 0.479 | 0.007 |
|  | BP | cell motility | GO:0048870 | 0.356 | 0.156 | 0.846 | | 0.806 | 0.447 | 0.008 |
|  | BP | cell projection organization | GO:0030030 | 0.660 | 0.359 | 0.743 | | 0.966 | 0.838 | 0.018 |
|  | CC | cell-cell junction | GO:0005911 | 0.005 | 0.018 | 0.016 | | 0.951 | 0.545 | 0.144 |
|  | CC | **contractile fiber** | GO:0043292 | 0.004 | 0.627 | 0.007 | | 0.186 | 0.135 | **<0.001** |
|  | BP | eye development | GO:0001654 | 0.239 | 0.205 | 0.327 | | 0.584 | 0.645 | 0.016 |
| Neuronal | CC | **fascia adherens** | GO:0005916 | **<0.001** | 0.240 | 0.026 | | 1.000 | 1.000 | 0.034 |
| signaling | CC | **I band** | GO:0031674 | 0.059 | 0.131 | 0.113 | | 0.572 | 0.032 | **<0.001** |
|  | CC | intercalated disc | GO:0014704 | 0.008 | 0.290 | 0.101 | | 1.000 | 1.000 | 0.082 |
|  | CC | **lamellipodium** | GO:0030027 | 1.000 | 0.012 | 0.003 | | 0.612 | 0.201 | **<0.001** |
|  | MF | **motor activity** | GO:0003774 | **<0.001** | 0.353 | 0.191 | | 0.721 | 0.949 | 0.010 |
|  | BP | muscle cell differentiation | GO:0042692 | 0.485 | 0.204 | 0.852 | | 0.946 | 0.973 | 0.024 |
|  | CC | **myofibril** | GO:0030016 | 0.004 | 0.615 | 0.006 | | 0.176 | 0.128 | **<0.001** |
|  | BP | neuron development | GO:0048666 | 0.264 | 0.177 | 0.414 | | 0.821 | 0.754 | 0.004 |
|  | BP | neuron projection development | GO:0031175 | 0.414 | 0.169 | 0.487 | | 0.875 | 0.660 | 0.005 |
|  | BP | neuron projection morphogenesis | GO:0048812 | 0.770 | 0.511 | 0.719 | | 0.889 | 0.834 | 0.040 |
|  | MF | protein tyrosine kinase activity | GO:0004713 | 0.037 | 1.000 | 0.780 | | 1.000 | 0.926 | 0.506 |
|  | CC | ruffle | GO:0001726 | 0.585 | 0.550 | 0.005 | | 1.000 | 0.499 | 0.091 |
|  | CC | sarcomere | GO:0030017 | 0.025 | 0.546 | 0.078 | | 0.132 | 0.093 | 0.004 |
|  | BP | sensory organ development | GO:0007423 | 0.459 | 0.259 | 0.647 | | 0.847 | 0.481 | 0.014 |
|  | BP | **tissue morphogenesis** | GO:0048729 | 0.186 | 0.126 | 0.639 | | 0.955 | 0.452 | **<0.001** |
|  | BP | vasculature development | GO:0001944 | 0.748 | 0.029 | 0.877 | | 0.966 | 0.628 | 0.173 |
|  | CC | **Z disc** | GO:0030018 | 0.044 | 0.124 | 0.088 | | 0.523 | 0.024 | **<0.001** |
| Post synaptic density | MF | nucleobase, nucleoside, nucleuotide kinase activity | GO:0019205 | 0.367 | 0.582 | 0.820 | | 0.027 | 0.627 | 0.377 |

MF: Molecular Function, CC: Cellular Components, BP: Biological Process. GO terms with *p* < 0.05 were listed in this table. GO terms with *p* < 0.001 were marked in bold.
