# Supplementary material for: Plausibility of the zebrafish embryos/larvae as an alternative animal model for autism: A comparison study of transcriptome changes
Source: PLoS One. 2018 Sep 4;13(9):e0203543. doi: 10.1371/journal.pone.0203543 (PMC6122816; doi:10.1371/journal.pone.0203543)
Supplement: S9 Table — (DOCX) [file pone.0203543.s011.docx]

**S9 Table. Significantly affected GO after VPA exposure in zebrafish embryo/larvae among the ASD related GOs in the BTBR mouse model suggested by Jasien *et al*. (2014)**

| **Tissue** | **Ontology** | **Term** | **GO ID** | ***p*-value (at 72 h)** | | | ***p*-value (at 120 h)** | | |
| --- | --- | --- | --- | --- | --- | --- | --- | --- | --- |
|  |  |  |  | **12.5** | **25** | **50** | **12.5** | **25** | **50** |
|  | CC | **cell leading edge** | GO:0031252 | 0.668 | 0.060 | **<0.001** | 0.864 | 0.539 | **<0.001** |
|  | CC | **cell projection** | GO:0042995 | 0.886 | 0.488 | **<0.001** | 0.715 | 0.522 | **<0.001** |
|  | CC | **cytoplasm** | GO:0005737 | **<0.001** | **<0.001** | **<0.001** | 0.813 | 0.053 | **<0.001** |
|  | CC | **cytoskeleton** | GO:0005856 | **<0.001** | 0.016 | **<0.001** | 0.799 | 0.665 | 0.001 |
|  | CC | **cytosol** | GO:0005829 | **<0.001** | **<0.001** | **<0.001** | 0.345 | 0.080 | **<0.001** |
| Cortical | CC | **filopodium** | GO:0030175 | 0.161 | 1.000 | 0.002 | 0.447 | 0.018 | **<0.001** |
|  | CC | **intracellular** | GO:0005622 | **<0.001** | 0.006 | 0.228 | 1.000 | 0.964 | **<0.001** |
|  | CC | **lamellipodium** | GO:0030027 | 1.000 | 0.012 | 0.003 | 0.612 | 0.201 | **<0.001** |
|  | CC | microtubule cytoskeleton | GO:0015630 | 0.012 | 0.109 | 0.655 | 0.975 | 0.923 | 0.009 |
|  | CC | **mitochondrion** | GO:0005739 | **<0.001** | 0.004 | **<0.001** | 0.698 | 0.009 | **<0.001** |
|  | CC | ruffle | GO:0001726 | 0.585 | 0.550 | 0.005 | 1.000 | 0.499 | 0.091 |
|  | BP | secretion by cell | GO:0032940 | 0.843 | 0.390 | 0.985 | 0.692 | 0.010 | 0.679 |
|  | BP | **transport** | GO:0006810 | 0.040 | 0.025 | **<0.001** | 0.088 | 0.003 | 0.069 |
|  | MF | actin binding | GO:0003779 | 0.439 | 0.387 | 0.005 | 0.507 | 0.410 | 0.032 |
|  | MF | actin filament binding | GO:0051015 | 0.812 | 1.000 | 0.006 | 1.000 | 1.000 | 0.418 |
|  | CC | axon | GO:0030424 | 0.601 | 0.802 | 0.276 | 0.543 | 0.467 | 0.002 |
|  | MF | calmodulin binding | GO:0005516 | 1.000 | 0.434 | 0.253 | 0.013 | 0.657 | 0.680 |
|  | MF | calmodulin-dependent protein kinase activity | GO:0004683 | 1.000 | 1.000 | 1.000 | 0.020 | 0.339 | 1.000 |
|  | MF | **catalytic activity** | GO:0003824 | 0.007 | **<0.001** | **<0.001** | **0.001** | **<0.001** | 0.029 |
| Hipocampal | CC | **cell projection** | GO:0042995 | 0.886 | 0.488 | **<0.001** | 0.715 | 0.522 | **<0.001** |
|  | BP | **cellular respiration** | GO:0045333 | **<0.001** | 0.002 | **<0.001** | 1.000 | 0.025 | **<0.001** |
|  | MF | coenzyme binding | GO:0050662 | 0.012 | 0.123 | 0.196 | 1.000 | 0.296 | 0.416 |
|  | MF | cofactor binding | GO:0048037 | 0.018 | 0.048 | 0.008 | 0.963 | 0.206 | 0.183 |
|  | CC | **cytoplasm** | GO:0005737 | **<0.001** | **<0.001** | **<0.001** | 0.813 | 0.053 | **<0.001** |
|  | CC | **cytoskeleton** | GO:0005856 | **<0.001** | 0.016 | **<0.001** | 0.799 | 0.665 | 0.001 |
|  | CC | **cytosol** | GO:0005829 | **<0.001** | **<0.001** | **<0.001** | 0.345 | 0.080 | **<0.001** |
|  | BP | generation of neurons | GO:0048699 | 0.183 | 0.097 | 0.751 | 0.795 | 0.820 | 0.001 |
|  | BP | **generation of precursor metabolites and energy** | GO:0006091 | 0.010 | 0.010 | **<0.001** | 0.066 | **<0.001** | **<0.001** |
|  | MF | GTPase activity | GO:0003924 | 0.070 | 0.035 | 0.007 | 0.440 | 0.749 | 0.005 |
|  | MF | **hydrogen ion transmembrane transporter activity** | GO:0015078 | **<0.001** | **<0.001** | **<0.001** | 0.874 | **<0.001** | **<0.001** |
|  | MF | **hydrolase activity, acting on acid anhydrides** | GO:0016817 | **<0.001** | 0.039 | **<0.001** | 0.700 | 0.452 | **<0.001** |
|  | MF | **hydrolase activity, acting on acid anhydrides, in phosphorus-containing anhydrides** | GO:0016818 | **<0.001** | 0.036 | **<0.001** | 0.683 | 0.434 | **<0.001** |
|  | CC | **intracellular** | GO:0005622 | **<0.001** | 0.006 | 0.228 | 1.000 | 0.964 | **<0.001** |
|  | CC | **intracellular membrane-bounded organelle** | GO:0043231 | 0.009 | 0.942 | 0.966 | 1.000 | 0.941 | 0.023 |
|  | CC | **intracellular organelle** | GO:0043229 | **<0.001** | 0.003 | 0.034 | 1.000 | 0.844 | **<0.001** |
| Hipocampal | CC | **mitochondrial inner membrane** | GO:0005743 | 0.001 | **<0.001** | **<0.001** | 0.632 | **0.001** | **0.000** |
|  | CC | **mitochondrial respiratory chain** | GO:0005746 | 0.004 | 0.003 | **<0.001** | 1.000 | **<0.001** | **<0.001** |
|  | CC | **mitochondrion** | GO:0005739 | **<0.001** | 0.004 | **<0.001** | 0.698 | 0.009 | **<0.001** |
|  | MF | **NADH dehydrogenase (ubiquinone) activity** | GO:0008137 | 0.004 | 0.048 | **<0.001** | 1.000 | 0.090 | 0.015 |
|  | MF | **NADH dehydrogenase activity** | GO:0003954 | 0.006 | 0.062 | **<0.001** | 1.000 | 0.108 | 0.022 |
|  | BP | **nervous system development** | GO:0007399 | 0.385 | 0.048 | 0.664 | 0.997 | 0.927 | **<0.001** |
|  | BP | neuron development | GO:0048666 | 0.264 | 0.177 | 0.414 | 0.821 | 0.754 | 0.004 |
|  | BP | neuron differentiation | GO:0030182 | 0.290 | 0.094 | 0.699 | 0.697 | 0.736 | 0.001 |
|  | BP | neuron projection development | GO:0031175 | 0.414 | 0.169 | 0.487 | 0.875 | 0.660 | 0.005 |
|  | BP | neuron projection morphogenesis | GO:0048812 | 0.770 | 0.511 | 0.719 | 0.889 | 0.834 | 0.040 |
|  | BP | neurotransmitter transport | GO:0006836 | 0.951 | 0.086 | 0.043 | 0.174 | 0.206 | 0.153 |
|  | MF | **nucleoside-triphosphatase activity** | GO:0017111 | **<0.001** | 0.020 | **<0.001** | 0.618 | 0.481 | **<0.001** |
|  | CC | organelle membrane | GO:0031090 | 0.445 | 0.106 | 0.157 | 0.788 | 0.562 | 0.007 |
|  | MF | **oxidoreductase activity** | GO:0016491 | **<0.001** | **<0.001** | **<0.001** | 0.687 | 0.004 | **<0.001** |
|  | MF | proline-rich region binding | GO:0070064 | 1.000 | 0.004 | 1.000 | 1.000 | 1.000 | 1.000 |
|  | CC | **protein complex** | GO:0043234 | <0.001 | 0.437 | 0.101 | 1.000 | 0.999 | 0.018 |
|  | MF | **pyrophosphatase activity** | GO:0016462 | <0.001 | 0.033 | **<0.001** | 0.676 | 0.427 | **<0.001** |
|  | CC | **respiratory chain** | GO:0070469 | **<0.001** | **<0.001** | **<0.001** | 1.000 | **<0.001** | **<0.001** |
|  | BP | **respiratory electron transport chain** | GO:0022904 | 0.031 | 0.003 | **<0.001** | 1.000 | 0.002 | 0.001 |
|  | BP | secretion by cell | GO:0032940 | 0.843 | 0.390 | 0.985 | 0.692 | 0.010 | 0.679 |
|  | MF | small molecule binding | GO:0036094 | 0.029 | 0.543 | 0.444 | 0.045 | 0.083 | 0.031 |
|  | BP | **small molecule metabolic process** | GO:0044281 | 0.004 | 0.051 | **<0.001** | 0.283 | 0.011 | 0.004 |
| Hipocampal | MF | syntaxin binding | GO:0019905 | 1.000 | 1.000 | 0.523 | 0.015 | 0.286 | 1.000 |
|  | MF | thioredoxin peroxidase activity | GO:0008379 | 1.000 | 1.000 | 1.000 | 1.000 | 0.027 | 1.000 |
|  | BP | **transport** | GO:0006810 | 0.040 | 0.025 | **<0.001** | 0.088 | 0.003 | 0.069 |
|  | CC | vesicle | GO:0031982 | 0.909 | 0.247 | 0.487 | 0.239 | 0.024 | 0.187 |

MF: Molecular Function, CC: Cellular Components, BP: Biological Process. GO terms with *p* < 0.05 were listed in this table. GO terms with *p* < 0.001 were marked in bold.
