# Supplementary material for: Plausibility of the zebrafish embryos/larvae as an alternative animal model for autism: A comparison study of transcriptome changes
Source: PLoS One. 2018 Sep 4;13(9):e0203543. doi: 10.1371/journal.pone.0203543 (PMC6122816; doi:10.1371/journal.pone.0203543)
Supplement: S10 Table — (DOCX) [file pone.0203543.s012.docx]

**S10 Table. The top 100 DEGs after 50 mM VPA exposure at 72 h based on *p*-value**

| Gene name | Description | *p*-value | Log_2_FC |
| --- | --- | --- | --- |
| *rpl24* | ribosomal protein L24 | 0.0001 | -3.19 |
| *rps15* | ribosomal protein S15 | 0.0001 | -3.36 |
| *ctrl* | chymotrypsin-like | 0.0001 | -5.07 |
| *prss59.1* | protease, serine, 59, tandem duplicate 1 | 0.0001 | -6.28 |
| *prss59.2* | protease, serine, 59, tandem duplicate 2 | 0.0001 | -4.82 |
| *fabp10a* | fatty acid binding protein 10a, liver basic | 0.0001 | -4.09 |
| *PKLR* | pyruvate kinase, liver and RBC | 0.0001 | -4.45 |
| *faua* | Finkel-Biskis-Reilly murine sarcoma virus (FBR-MuSV) ubiquitously expressed a | 0.0001 | -3.63 |
| *CEL* | Bile salt-activated lipase | 0.0001 | -3.76 |
| *CPB1* | Carboxypeptidase B | 0.0001 | -4.44 |
| *ctrb1* | chymotrypsinogen B1 | 0.0001 | -5.25 |
| *rpl14* | ribosomal protein L14 | 0.0001 | -3.61 |
| *crygn2* | crystallin, gamma N2 | 0.0001 | -3.87 |
| *lgals2b* | lectin, galactoside-binding, soluble, 2b | 0.0001 | -2.95 |
| *afp4* | antifreeze protein type IV | 0.0001 | -3.16 |
| *ELA1_SALSA* | Elastase-1 | 0.0001 | -4.1 |
| *h3f3d* | H3 histone, family 3D | 0.0001 | -2.94 |
| *CPA1* | Carboxypeptidase A1 | 0.0001 | -3.55 |
| *fndc7b* | fibronectin type III domain containing 7b | 0.0002 | -2.44 |
| *rplp2l* | ribosomal protein, large P2, like | 0.0002 | -2.79 |
| *rho* | rhodopsin | 0.0002 | -4.09 |
| *krt15* | keratin 15 | 0.0003 | -2.8 |
| *slc6a19a.2* | solute carrier family 6 (neutral amino acid transporter), member 19a, tandem duplicate 2 | 0.0003 | -3.63 |
| *mpz* | myelin protein zero | 0.0003 | -5.68 |
| *cox6c* | cytochrome c oxidase subunit VIc | 0.0003 | -2.71 |
| *cox6a1* | cytochrome c oxidase subunit VIa polypeptide 1 | 0.0003 | -4.37 |
| *pdia2* | protein disulfide isomerase family A, member 2 | 0.0003 | -2.99 |
| *rpl31* | ribosomal protein L31 | 0.0003 | -3.81 |
| *Cfd* | Complement factor D | 0.0004 | -4.27 |
| *rpl13* | ribosomal protein L13 | 0.0004 | -3.08 |
| *ddx39ab* | DEAD (Asp-Glu-Ala-Asp) box polypeptide 39Ab | 0.0005 | -2.86 |
| *atp5l* | ATP synthase, H+ transporting, mitochondrial F0 complex, subunit g | 0.0006 | -4.21 |
| *ppiab* | peptidylprolyl isomerase Ab (cyclophilin A) | 0.0006 | -2.81 |
| *chia.2* | chitinase, acidic.2 | 0.0006 | -2.77 |
| *amy2a* | amylase, alpha 2A (pancreatic) | 0.0006 | -4.35 |
| *gngt2b* | guanine nucleotide binding protein (G protein), gamma transducing activity polypeptide 2b | 0.0007 | -2.54 |
| *arf4b* | ADP-ribosylation factor 4b | 0.0008 | 4.46 |
| *rps26l* | ribosomal protein S26, like | 0.0008 | -4.03 |
| *tm4sf4* | transmembrane 4 L six family member 4 | 0.0008 | -3.06 |
| *rps3* | ribosomal protein S3 | 0.0008 | -2.98 |
| *ppp1cbl* | protein phosphatase 1, catalytic subunit, beta isoform, like | 0.0008 | -3.81 |
| *cpa4* | carboxypeptidase A4 | 0.0009 | -3.45 |
| *pvalb8* | parvalbumin 8 | 0.0009 | -2.67 |
| *YH24_CAEEL* | Putative aminopeptidase W07G4.4 | 0.0009 | -2.29 |
| *acta2* | actin, alpha 2, smooth muscle, aorta | 0.001 | -2.68 |
| *rps28* | ribosomal protein S28 | 0.001 | -3.01 |
| *ace2* | angiotensin I converting enzyme 2 | 0.0011 | -2.54 |
| *rpl28* | ribosomal protein L28 | 0.0011 | -2.86 |
| *pdzk1* | PDZ domain containing 1 | 0.0012 | -2.81 |
| *tuba8l2* | tubulin, alpha 8 like 2 | 0.0013 | -3.06 |
| *uqcrfs1* | ubiquinol-cytochrome c reductase, Rieske iron-sulfur polypeptide 1 | 0.0013 | -2.5 |
| *vil1* | villin 1 | 0.0013 | -2.29 |
| *Evpl* | Envoplakin | 0.0014 | -3.38 |
| *rps6* | ribosomal protein S6 | 0.0015 | -3.28 |
| *rps16* | ribosomal protein S16 | 0.0015 | -2.77 |
| *rplp2* | ribosomal protein, large P2 | 0.0015 | -2.26 |
| *bbox1* | butyrobetaine (gamma), 2-oxoglutarate dioxygenase (gamma-butyrobetaine hydroxylase) 1 | 0.0015 | -2.94 |
| *cyp7a1* | cytochrome P450, family 7, subfamily A, polypeptide 1 | 0.0016 | -3.21 |
| *hsd17b4* | hydroxysteroid (17-beta) dehydrogenase 4 | 0.0016 | -3.03 |
| *pdlim7* | PDZ and LIM domain protein 7 | 0.0016 | -2.57 |
| *muc5.3* | mucin 5.3 | 0.0018 | -2.43 |
| *acsl1b* | acyl-CoA synthetase long-chain family member 1b | 0.0019 | -2.31 |
| *RPS20* | 40S ribosomal protein | 0.0019 | -2.76 |
| *CABZ01075268.2* | T-complex protein 1 subunit beta | 0.0019 | -2.44 |
| *hephl1b* | hephaestin-like 1b | 0.002 | 3.17 |
| *mag* | myelin associated glycoprotein | 0.0021 | -2.34 |
| *atp5j* | ATP synthase, H+ transporting, mitochondrial Fo complex, subunit F6 | 0.0022 | -2.71 |
| *plac8.1* | placenta-specific 8, tandem duplicate 1 | 0.0022 | -3.04 |
| ***ak1*** | **adenylate kinase 1** | **0.0023** | **-2.64** |
| *wdr1* | WD repeat domain 1 | 0.0024 | -2.78 |
| *mybpc2a* | myosin binding protein C, fast type a | 0.0024 | -2.61 |
| *epd* | ependymin | 0.0024 | -2.77 |
| *zanl* | zonadhesin, like | 0.0024 | -2.3 |
| *fabp1b.1* | fatty acid binding protein 1b, tandem duplicate 1 | 0.0024 | -2.44 |
| *wdr1-a* | WD repeat-containing protein 1-A | 0.0024 | -3.81 |
| *irbp* | interphotoreceptor retinoid-binding protein | 0.0026 | -2.17 |
| *SI* | sucrase-isomaltase | 0.0026 | -2.39 |
| *CF058_DANRE* | UPF0762 protein C6orf58 homolog | 0.0026 | -3.02 |
| *aldocb* | aldolase C, fructose-bisphosphate, b | 0.0026 | -2.4 |
| *rpl23* | ribosomal protein L23 | 0.0026 | -2.63 |
| *ndufb9* | NADH dehydrogenase (ubiquinone) 1 beta subcomplex, 9 | 0.0027 | -2.28 |
| *tnni2a.1* | troponin I type 2a (skeletal, fast), tandem duplicate 1 | 0.0027 | -2.24 |
| *cox7c* | cytochrome c oxidase, subunit VIIc | 0.0027 | -2.2 |
| *smyd1a* | SET and MYND domain containing 1a | 0.0027 | -2.4 |
| *timm8b* | translocase of inner mitochondrial membrane 8 homolog B (yeast) | 0.0029 | -3.63 |
| *mt2* | metallothionein 2 | 0.003 | -2.21 |
| *ctsl.1* | cathepsin L.1 | 0.0031 | -2.7 |
| *sdhb* | succinate dehydrogenase complex, subunit B, iron sulfur (Ip) | 0.0031 | -2.35 |
| *slc25a3b* | solute carrier family 25 (mitochondrial carrier; phosphate carrier), member 3b | 0.0031 | -2.6 |
| *cyp8b1* | cytochrome P450, family 8, subfamily B, polypeptide 1 | 0.0032 | -2.55 |
| *tmem86b* | transmembrane protein 86B | 0.0032 | -2.03 |
| *ela3l* | elastase 3 like | 0.0032 | -6.12 |
| *zgc:114174* | zgc:114174 | 0.0034 | -2.47 |
| *sepm* | Selenoprotein M | 0.0034 | -3.57 |
| *s100a10a* | S100 calcium binding protein A10a | 0.0035 | -3.69 |
| *irf2bp2a* | interferon regulatory factor 2 binding protein 2a | 0.0036 | -2.72 |
| *cyp2x9* | cytochrome P450, family 2, subfamily X, polypeptide 9 | 0.0037 | -3.36 |
| *zgc:165423* | zgc:165423 | 0.0037 | -2.37 |
| *pvalb4* | parvalbumin 4 | 0.0037 | -2.55 |
| *rps13* | ribosomal protein S13 | 0.0037 | -2.39 |

DEGs related to ASD were marked in bold.
