# Supplementary material for: Plausibility of the zebrafish embryos/larvae as an alternative animal model for autism: A comparison study of transcriptome changes
Source: PLoS One. 2018 Sep 4;13(9):e0203543. doi: 10.1371/journal.pone.0203543 (PMC6122816; doi:10.1371/journal.pone.0203543)
Supplement: S11 Table — (DOCX) [file pone.0203543.s013.docx]

**S11 Table. The top 100 DEGs after 50 mM VPA exposure at 120 h based on *p*-value**

| **Gene** | **Description** | ***p*-value** | **Log_2_FC** |
| --- | --- | --- | --- |
| *h3f3d* | H3 histone, family 3D | 0.0001 | 4.22 |
| *RPS20* | 40S ribosomal protein S20 | 0.0002 | 3.71 |
| *irf2bp2a* | interferon regulatory factor 2 binding protein 2a | 0.0003 | 3.93 |
| *rpl14* | ribosomal protein L14 | 0.0003 | 3.76 |
| *crygn2* | crystallin, gamma N2 | 0.0003 | 3.8 |
| *rpl31* | ribosomal protein L31 | 0.0003 | 4.7 |
| *rplp2l* | ribosomal protein, large P2, like | 0.0004 | 3.54 |
| *sid4* | secreted immunoglobulin domain 4 | 0.0006 | 4.04 |
| *rps26l* | ribosomal protein S26, like | 0.0006 | 4.74 |
| *CABZ01112656.1* | Growth hormone-inducible transmembrane protein | 0.0008 | 5.13 |
| *faua* | Finkel-Biskis-Reilly murine sarcoma virus (FBR-MuSV) ubiquitously expressed a | 0.0008 | 3.69 |
| *CABZ01108998.1* | Protein disulfide-isomerase TMX3 | 0.0008 | 6.12 |
| *uqcrfs1* | ubiquinol-cytochrome c reductase, Rieske iron-sulfur polypeptide 1 | 0.0009 | 3.04 |
| *ppp1cbl* | protein phosphatase 1, catalytic subunit, beta isoform, like | 0.0009 | 3.69 |
| *cox6a1* | cytochrome c oxidase subunit VIa polypeptide 1 | 0.0009 | 4.06 |
| *timp2b* | TIMP metallopeptidase inhibitor 2b | 0.001 | 3.88 |
| *rplp2* | ribosomal protein, large P2 | 0.001 | 3.08 |
| *gart* | phosphoribosylglycinamide formyltransferase | 0.0011 | 3.51 |
| *matn1* | matrilin 1 | 0.0011 | 3.21 |
| *cox8a* | cytochrome c oxidase subunit VIIIA (ubiquitous) | 0.0011 | 3.37 |
| *rps12* | ribosomal protein S12 | 0.0012 | 5.11 |
| *atp5j* | ATP synthase, H+ transporting, mitochondrial Fo complex, subunit F6 | 0.0013 | 3.08 |
| *ppiab* | peptidylprolyl isomerase Ab (cyclophilin A) | 0.0013 | 3.44 |
| *fkbp5* | FK506 binding protein 5 | 0.0013 | 3.48 |
| *slc25a20* | solute carrier family 25 (carnitine/acylcarnitine translocase), member 20 | 0.0017 | 3.50 |
| *rpl28* | ribosomal protein L28 | 0.0019 | 3.11 |
| *rpl13* | ribosomal protein L13 | 0.0019 | 3.67 |
| *ddx39ab* | DEAD (Asp-Glu-Ala-Asp) box polypeptide 39Ab | 0.002 | 3.06 |
| *CABZ01092156.1* | Myelin protein zero-like protein 3 | 0.002 | 3.92 |
| *atp5g3a* | ATP synthase, H+ transporting, mitochondrial Fo complex, subunit C3 (subunit 9), genome duplicate a | 0.0021 | 2.85 |
| *sp1* | sp1 transcription factor | 0.0024 | 4.94 |
| ***ddb1*** | **damage-specific DNA binding protein 1** | **0.0024** | **3.55** |
| *rps16* | ribosomal protein S16 | 0.0027 | 2.98 |
| *rps15* | ribosomal protein S15 | 0.0029 | 3.82 |
| *hbbe2* | hemoglobin beta embryonic-2 | 0.003 | 3.54 |
| *daglb* | diacylglycerol lipase, beta | 0.0032 | 2.73 |
| *rab3gap1* | Rab3 GTPase-activating protein catalytic subunit | 0.0033 | 3.89 |
| *acta1b* | actin, alpha 1b, skeletal muscle | 0.0034 | 3.72 |
| *odc1* | ornithine decarboxylase 1 | 0.0034 | 3.01 |
| *crybb1* | crystallin, beta B1 | 0.0036 | 3.09 |
| *CABZ01075268.2* | T-complex protein 1 subunit beta | 0.0036 | 2.91 |
| *zgc:65894* | zgc:65894 | 0.0037 | 2.93 |
| *wdr1* | WD repeat domain 1 | 0.0039 | 3.40 |
| *rpl24* | ribosomal protein L24 | 0.0042 | 3.27 |
| *DYNC1H1* | dynein cytoplasmic 1 heavy chain 1 | 0.0042 | 3.56 |
| ***ak1*** | **adenylate kinase 1** | **0.0044** | **3.22** |
| *STXBP6* | syntaxin binding protein 6 | 0.0047 | 3.40 |
| *col9a2* | procollagen, type IX, alpha 2 | 0.0047 | 2.58 |
| *ndufb11* | NADH dehydrogenase (ubiquinone) 1 beta subcomplex, 11 | 0.0047 | 3.15 |
| *col9a3* | collagen, type IX, alpha 3 | 0.0048 | 2.54 |
| *tmem39a* | transmembrane protein 39A | 0.0049 | 7.23 |
| *fabp1b.1* | fatty acid binding protein 1b, tandem duplicate 1 | 0.0049 | 2.75 |
| *cox7c* | cytochrome c oxidase, subunit VIIc | 0.0051 | 2.56 |
| *ndufb9* | NADH dehydrogenase (ubiquinone) 1 beta subcomplex, 9 | 0.0052 | 2.38 |
| *Evpl* | Envoplakin [Source:SWISS;Acc:Q9D952] | 0.0052 | 3.12 |
| *LAMP5* | lysosomal associated membrane protein family member 5 | 0.0053 | 4.63 |
| *harbi1* | Putative nuclease HARBI1 | 0.0054 | 3.01 |
| *apoa1a* | apolipoprotein A-Ia | 0.0054 | 3.00 |
| *hspb1* | heat shock protein, alpha-crystallin-related, 1 | 0.0056 | 2.84 |
| *PLB* | Putative phospholipase B 81b | 0.0057 | 2.4 |
| *atp5d* | ATP synthase, H+ transporting, mitochondrial F1 complex, delta subunit | 0.0059 | 6.14 |
| *pol* | RNA-directed DNA polymerase from mobile element jockey | 0.006 | 5.51 |
| *rps28* | ribosomal protein S28 | 0.0061 | 3.25 |
| *PKLR* | pyruvate kinase, liver and RBC | 0.0062 | 3.44 |
| *DES* | Desmin | 0.0062 | 4.67 |
| *PTPRS* | Receptor-type tyrosine-protein phosphatase S | 0.0063 | 4.12 |
| *txn2* | thioredoxin 2 | 0.0063 | 3.03 |
| *fubp3* | far upstream element (FUSE) binding protein 3 | 0.0063 | 4.12 |
| *nppa* | natriuretic peptide A | 0.0063 | 3.38 |
| *slc6a19a.1* | solute carrier family 6 (neutral amino acid transporter), member 19a, tandem duplicate 1 | 0.0064 | 2.69 |
| *cebpb* | CCAAT/enhancer binding protein (C/EBP), beta | 0.0064 | 4.52 |
| *ndufb6* | NADH dehydrogenase (ubiquinone) 1 beta subcomplex, 6 | 0.0065 | 2.91 |
| *gatm* | glycine amidinotransferase (L-arginine:glycine amidinotransferase) | 0.0067 | 5.08 |
| *rps3* | ribosomal protein S3 | 0.0067 | 2.83 |
| *timm8b* | translocase of inner mitochondrial membrane 8 homolog B (yeast) | 0.0067 | 4.65 |
| *ACTN3* | Alpha-actinin-3 | 0.0069 | 5.80 |
| *TNS3* | Tensin-3 | 0.007 | 3.27 |
| *mcm2* | minichromosome maintenance complex component 2 | 0.007 | 2.85 |
| *wdr1-a* | WD repeat-containing protein 1-A | 0.007 | 4.47 |
| *SNTX subunit beta* | Stonustoxin subunit beta | 0.0071 | -2.56 |
| *YB039_MOUSE* | Uncharacterized protein LINC00116 homolog | 0.0074 | 2.96 |
| *cpa4* | carboxypeptidase A4 | 0.0074 | -2.64 |
| *si:ch211-270n8.1* | si:ch211-270n8.1 | 0.0076 | 4.93 |
| *zgc:92590* | zgc:92590 | 0.0076 | -3.25 |
| *si:dkey-248g15.3* | si:dkey-248g15.3 | 0.0076 | 2.81 |
| *CABZ01103941.1* | Tensin-3 | 0.0077 | 3.61 |
| *NCSTN* | nicastrin | 0.008 | 3.03 |
| *CABZ01102240.1* | NADH dehydrogenase [ubiquinone] 1 alpha subcomplex subunit 13 | 0.008 | 5.29 |
| *slc25a10* | solute carrier family 25 (mitochondrial carrier; dicarboxylate transporter), member 10 | 0.0081 | 3.87 |
| *col9a1a* | collagen, type IX, alpha 1a | 0.0081 | 2.34 |
| *adamts1* | ADAM metallopeptidase with thrombospondin type 1 motif, 1 [ | 0.0082 | 2.9 |
| *ndufc2* | NADH dehydrogenase (ubiquinone) 1, subcomplex unknown, 2 | 0.0083 | 2.96 |
| *mcm7* | minichromosome maintenance complex component 7 | 0.0085 | 5.28 |
| *si:ch211-55p10.3* | si:ch211-255p10.3 | 0.0086 | 2.65 |
| *ndufs5* | NADH dehydrogenase (ubiquinone) Fe-S protein 5 [ | 0.0086 | 4.20 |
| *mmp9* | matrix metallopeptidase 9 | 0.0086 | 2.53 |
| *acadsb* | acyl-CoA dehydrogenase, short/branched chain | 0.0089 | 3.2 |
| *col2a1b* | collagen, type II, alpha 1b | 0.0092 | 5.80 |
| *ACTC1* | zgc:86709 | 0.0094 | 2.67 |
| *rpl18a* | ribosomal protein L18a | 0.0096 | 2.45 |

DEGs related to ASD were marked in bold.
